# Indoprofen prevents muscle wasting in aged mice through activation of PDK1/AKT pathway

**DOI:** 10.1002/jcsm.12558

**Published:** 2020-02-25

**Authors:** Hyebeen Kim, Sung Chun Cho, Hyeon‐Ju Jeong, Hye‐Young Lee, Myong‐Ho Jeong, Jung‐Hoon Pyun, Dongryeol Ryu, MinSeok Kim, Young‐Sam Lee, Minseok S. Kim, Sang Chul Park, Yun‐Il Lee, Jong‐Sun Kang

**Affiliations:** ^1^ Department of Molecular Cell Biology, Single Cell Network Research Center Sungkyunkwan University Suwon South Korea; ^2^ Samsung Biomedical Research Institute Samsung Medical Center Seoul South Korea; ^3^ Well Aging Research Center, Division of Biotechnology Daegu Gyeongbuk Institute of Science and Technology (DGIST) Daegu South Korea; ^4^ School of Undergraduate Studies Daegu Gyeongbuk Institute of Science and Technology (DGIST) Daegu South Korea; ^5^ Department of New Biology Daegu Gyeongbuk Institute of Science and Technology (DGIST) Daegu South Korea

**Keywords:** Exercise mimetic, Hypertrophic response, Indoprofen, Muscle wasting, Muscle weakness, PDK1

## Abstract

**Background:**

Muscle wasting, resulting from aging or pathological conditions, leads to reduced quality of life, increased morbidity, and increased mortality. Much research effort has been focused on the development of exercise mimetics to prevent muscle atrophy and weakness. In this study, we identified indoprofen from a screen for peroxisome proliferator‐activated receptor γ coactivator α (PGC‐1α) inducers and report its potential as a drug for muscle wasting.

**Methods:**

The effects of indoprofen treatment on dexamethasone‐induced atrophy in mice and in 3‐phosphoinositide‐dependent protein kinase‐1 (PDK1)‐deleted C2C12 myotubes were evaluated by immunoblotting to determine the expression levels of myosin heavy chain and anabolic‐related and oxidative metabolism‐related proteins. Young, old, and disuse‐induced muscle atrophic mice were administered indoprofen (2 mg/kg body weight) by gavage. Body weight, muscle weight, grip strength, isometric force, and muscle histology were assessed. The expression levels of muscle mass‐related and function‐related proteins were analysed by immunoblotting or immunostaining.

**Results:**

In young (3‐month‐old) and aged (22‐month‐old) mice, indoprofen treatment activated oxidative metabolism‐related enzymes and led to increased muscle mass. Mechanistic analysis using animal models and muscle cells revealed that indoprofen treatment induced the sequential activation of AKT/p70S6 kinase (S6K) and AMP‐activated protein kinase (AMPK), which in turn can augment protein synthesis and PGC‐1α induction, respectively. Structural prediction analysis identified PDK1 as a target of indoprofen and, indeed, short‐term treatment with indoprofen activated the PDK1/AKT/S6K pathway in muscle cells. Consistent with this finding, PDK1 inhibition abrogated indoprofen‐induced AKT/S6K activation and hypertrophic response.

**Conclusions:**

Our findings demonstrate the effects of indoprofen in boosting skeletal muscle mass through the sequential activation of PDK1/AKT/S6K and AMPK/PGC‐1α. Taken together, our results suggest that indoprofen represents a potential drug to prevent muscle wasting and weakness related to aging or muscle diseases.

## Introduction

Sarcopenia is a characterized by progressive loss of skeletal muscle mass and strength related with aging, resulting in a decline of muscle function and an increased risk of developing various chronic diseases.^1^, ^2^ In sarcopenic patients, the physical activity improves neuromuscular activity, reduces inflammation, and enhances muscle function.^3^ Accumulating evidence suggests that peroxisome proliferator‐activated receptor γ coactivator α (PGC‐1α) is a key target induced by exercise.^4^, ^5^ PGC‐1α, as a transcriptional coactivator, evokes the expression of genes associated with exercise, including genes involved in mitochondrial biogenesis, stimulation of fatty acid oxidation, angiogenesis, and resistance to muscle atrophy.^6^ Multiple studies have reported the beneficial effects of elevated PGC‐1α levels for the prevention of aging‐related metabolic diseases and sarcopenia.^3^, ^7^, ^8^ The AMP‐activated protein kinase (AMPK) pathway is a crucial sensor of the energy status that induces and activates PGC‐1α. Consistently, the AMPK agonists AICAR and metformin have been shown to induce PGC‐1α expression and mimic the effects of exercise on muscle strength.^9^, ^10^, ^11^

In addition to PGC‐1α, a network of interconnected signals involved in protein synthesis and protein degradation controls the maintenance of muscle mass and strength.^8^ Among anabolic stimuli, insulin‐like growth factor 1 (IGF1) and a cascade of intracellular effectors including phosphatidylinositol‐3‐kinase (PI3K), 3‐phosphoinositide‐dependent protein kinase‐1 (PDK1), AKT, the mammalian target of rapamycin (mTOR), and S6K activate protein synthesis thereby contribute to muscle growth.^12^ The significance of boosting anabolic pathway to prevent sarcopenia has been underlined by several studies.^13^, ^14^, ^15^ Thus, targeting anabolic pathways such as IGF‐1 or its downstream signalling seems to be an attractive strategy to develop therapeutics to prevent sarcopenia or other muscle atrophy conditions. Supporting this idea, the genetic activation of IGF‐1 or AKT in skeletal muscle is sufficient to increase muscle mass.^16^ The activation of AKT seems to be an effective way to enhance muscle mass, as AKT controls protein synthesis through mTOR/S6K activation and protein degradation by repressing the transcription factor FOXO and muscle‐specific ubiquitin ligases MAFbx/Atrogin‐1 and MuRF1.^16^, ^17^ AKT can be phosphorylated on distinct residues by kinases like PDK1 and the mTOR‐Rictor complex.^18^, ^19^ Overall, PDK1 functions as a master kinase, phosphorylating and activating AKT, S6K, and ribosomal S6 kinase.^20^, ^21^, ^22^ Resistance exercise has been shown to sequentially activate PDK1/AKT/S6K and AMPK/PGC‐1α. These proteins are important for protein synthesis and modulation of oxidative muscle metabolism, through which they can boost muscle size and strength.^23^

In this study, we examined the effects of a nonsteroidal anti‐inflammatory drug, indoprofen, identified as a PGC‐1α inducer from a high‐throughput screen in C2C12 muscle cells. Indoprofen treatment improved muscle strength and muscle mass in aged mice and in muscle atrophy mouse model. Our data demonstrate that indoprofen activated the AKT/S6K pathway, as well as PGC‐1α, to promote muscle protein synthesis and inhibit protein degradation, likely contributing to increased muscle mass in aged mice and the muscle atrophy models. Structural prediction analysis identified PDK1 as a target of indoprofen. Consistent with this possibility, PDK1 depletion in skeletal muscle abrogated the hypertrophic effect induced by indoprofen. Altogether, our study suggests that indoprofen has great potential as a drug to prevent muscle loss and weakness associated with aging or pathological conditions.

## Materials and methods

### Mice

Wild‐type C57BL/6 J male mice were obtained from Korea Basic Science Institute and maintained until use. All mice were housed at 23°C with a 12:12 h light–dark cycle with *ad libitum* access to food and water. The mice were orally administered a daily dose of 2 mg/kg indoprofen for 4–8 weeks (3‐month‐old mice, young) or 12 weeks (22‐month‐old mice, old) to assess the effect of indoprofen, and control mice were administered the same amount of vehicle dimethyl sulphoxide (DMSO) dissolved in saline. For the muscle atrophy experiment, the 3‐month‐old mice were orally administered either vehicle or indoprofen for 1 week prior to hanging suspension for 2 weeks in an environment with *ad libitum* access to food and water. All animals were sacrificed following 16 h of fasting, and their muscles were harvested 4 h after the last administration of indoprofen. All animal experiments were approved by the Institutional Animal Care and Research Advisory Committee at Sungkyunkwan University School of Medicine, Laboratory Animal Research Center and complied with the regulations of the institutional ethics committee.

### Cell culture, luciferase assay, and puromycin incorporation assay

C2C12 myoblasts were maintained as previously described.^24^ They were cultured in growth medium (Dulbecco's Modified Eagle Medium high glucose, Gibco) containing 15% foetal bovine serum (Gibco), 10 units per millimetre penicillin, and 10 μg/mL streptomycin (Welgene Inc.) at 37°C, 5% CO_2_. Differentiation medium contained (Dulbecco's Modified Eagle Medium high glucose) 2% horse serum, 10 units per millimetre of penicillin, and 10 μg/mL of streptomycin.

Luciferase assay was performed as previously described.^25^ C2C12 cells were transfected with an expression plasmid for luciferase responsive to the 2 kb promoter region of PGC‐1α (Addgene plasmid #8887), PGC‐1α promoter delta CRE site (Addgene plasmid #8888), and PGC‐1α promoter delta MEF2 site (Addgene plasmid #8889) using Lipofectamine 2000 (Invitrogen) according to the manufacturer's instructions. After 24 h of transfection, the cells were incubated in differentiation media with a chemical library (Prestwick Chemical) for high‐throughput screening and indoprofen. The cells were lysed with Reporter Lysis Buffer (Promega), and luciferase assays were performed using a Luciferase Assay System kit and a luminometer (Berthold Technologies). The transfection efficiency was normalized based on the cotransfected β‐galactosidase enzyme activity measured using an assay kit (Promega).

The protein synthesis analysis was carried out by using SUnSET method.^26^, ^27^ C2C12 cells were treated with 10 μg/mL puromycin for 1 h, followed by puromycin incorporation assessment by immunoblotting.

### Western blotting

Cells and muscle tissue were dissolved in radioimmunoprecipitation buffer [1 × phosphate‐buffered saline, 1% IGEPAL CA‐630 (v/v), 0.1% sodium dodecyl sulphate (SDS) (w/v), and 0.5% sodium deoxycholate (w/v), and 50 mM sodium fluoride]. The lysates (20 μg) were resolved on SDS‐PAGE and electrotransfered to polyvinylidene difluoride membranes. We used the following antibodies for western blotting: Myh (MF‐20, 1:1,000; Developmental Studies Hybridoma Bank), PGC‐1α (ST1202, 1:1000), Puromycin (MABE343, 1:20 000), p‐IRS1 (Ser307) (07‐247, 1:1000), IRS1 (06‐248, 1:1000) (Millipore), p‐AKT [Ser473 (#4058, 1:1000), Thr308 (#9275, 1:500)], AKT (#9272, 1:1000), p‐PDK1 [Ser241 (#3062, 1:1000)], PDK1 (#3061, 1:1,000), phosphorylated (active) AMPK (p‐AMPK) Thr172 (#2535, 1:500), AMPK (#5831, 1:1000), p‐mTOR [Ser2448 (#2971, 1:1000)], mTOR (#2972, 1:1000), p‐S6K [Thr389 (#9205, 1:1000)], S6K (#9202, 1:1000), p‐PI3K p85/p55 (Tyr458/Tyr199) (#4228, 1:1000), PI3K (#4257, 1:1000), p‐cAMP response element‐binding protein (CREB) (Ser133) (#9198, 1:1000), CREB (#9197, 1:1000), p‐FOXO1 (Ser256) (#9461, 1:1000), FOXO1 (#2880, 1:1000) (Cell Signaling Technology), Myoglobin (ab77232, 1:1000), T‐OXPHOS (ab110413, 1:1000) (Abcam), β‐tubulin (t5293, 1:1000; Sigma), Atrogin‐1 (sc‐33782, 1:1000), HSP90 (sc‐7947, 1:1000), p‐ATF2 (Thr71) (sc‐8398, 1:1000), activating transcription factor‐2 (ATF2) (sc‐187, 1:1000) (Santa Cruz Biotechnology), MuRF1 (GTX110475, 1:1000; GeneTex), GAPDH (#LF‐PA0018, 1:5000; AbFrontier), and Cytochrome C (556433, 1:1000; BD Biosciences). For the calculation of relative phosphorylation levels, the densitometries of the immunoblots of the phospho‐Abs were normalized to the total protein levels in each experiment; the averages of the relative levels of phosphorylation in more than three independent experiments have been presented.

### Cryosections, staining analysis, and fibre size measurement

Muscle tissue was embedded in Tissue‐Tek OCT Compound (Sakura), and 7 μm thick serial sections for staining were cut using a cryomicrotome. To analyse the NADH dehydrogenase activity, we incubated the sections in 0.9 mM NADH and 1.5 mM nitro blue tetrazolium (Sigma) in 3.5 mM phosphate buffer (pH 7.4) for 30 min. To analyse the succinate dehydrogenase (SDH) activity, we incubated the sections for 1 h in 50 μM sodium succinate and 0.3 mM nitro blue tetrazolium in 114 mM phosphate buffer containing K‐EGTA (Sigma). Myosin heavy chain (Myh) immunostaining of muscle tissue sections was performed in the order of fixation, permeation, and incubation with primary antibodies against MyhI, MyhIIa, MyhIIb (Developmental Studies Hybridoma Bank), and laminin (Abcam). For Myh immunostaining, MyhIIa (BF‐32, 1:200), MyhIIb (BF‐F3, 1:200) (Developmental Studies Hybridoma Bank), and laminin (ab11575, 1:200; Abcam) antibodies were used. The images were captured and processed with a Nikon ECLIPSE TE‐2000 U inverted microscope using nis‐elements f software (Nikon), and the myofibre area was measured using imagej software. For muscle histology, the cryosections were stained with Mayer's hematoxylin and eosin (BBC Biomedical). The images were captured using a Nikon ECLIPS TE‐2000 U inverted microscope or tissue faxs imaging software (TissueGnostics).

### RNA isolation and quantitative polymerase chain reaction

Quantitative real‐time polymerase chain reaction (qRT‐PCR) analysis was performed as previously described.^28^ Briefly, the tissues were homogenized by FastPrepR‐24 (MP Biomedicals) and extracted using an easy‐spin Total RNA Extraction Kit (iNtRON Biotechnology). The fold change in gene expression was normalized against the expression of ribosomal gene L32. The sequences of the primers used in this study are provided in Supporting Information, *Table* *S1*.

### Treadmill running exercise and grip strength test

A Columbus Exer‐6 M treadmill was used for treadmill running tests. Prior to the tests, the mice were acclimatized to the treadmill with a daily 5 min run at 7 m/min for 5 days. The treadmill test was performed at 9 m/min for 30 min, followed by an increase up to 9 m/min until exhaustion. Grip strength was measured using a grip strength metre (Bioseb). The animal was allowed to grab the grid with a forelimb and pulled gently with consistent force until the forelimb was detached from the grid. The maximal strength was recorded when the forelimb detached from the grid. Three trials were performed on each animal. Experimenters were blind to the treatment conditions of the mice during testing.

### Isometric force

The muscle contractile properties of the soleus (SOL) and extensor digitorum longus (EDL) muscles isolated from old‐control (25‐month‐old), old‐indoprofen‐treated mice were examined. The isolated muscles were transferred to a chamber containing Krebs‐Ringer buffer (118 mM NaCl, 4.75 mM KCl, 24.8 mM NaHCO_3_, 1.18 mM KH_2_PO_4_, 2.5 mM CaCl_2_, 1.18 mM MgSO_4_, and 10 mM glucose, pH 7.4) and oxygenated with 95% O_2_ and 5% CO_2_ while being thermostatically maintained at 25°C. The proximal tendon was fixed using a clamp, and the distal tendon was fixed to a force transducer (Grass Instruments, FT.03, USA). The optimum muscle length was determined as the length producing the highest twitch force (mN) with a supramaximal pulse of 0.2 ms duration. Isometric tetanic contractions (mN) were determined using an electrical stimulator (Grass Instruments, S48) at a stimulation frequency of 150 Hz for the SOL and 100 Hz for the EDL muscle, a stimulation current of 100 V, for a duration of 800 ms (SOL) or 200 ms (EDL). Then, SOL muscle fatigue was analysed in Krebs–Ringer buffer using a repeated electrical stimulation protocol. The muscles were given a 1 s, 150 Hz tetanus every 2 s over a period of 30 s, and the fatigue index was analysed as the difference from the first contractile force and plotted as a fatigue curve.

### 3‐Phosphoinositide‐dependent protein kinase‐1 expression, purification, and *in vitro* activity test

The maltose‐binding protein (MBP)‐tagged protein kinase domain of PDK1 (55–361 amino acids) was expressed in HEK293 cells and purified through an amylose resin column (New England Biolabs) and a Superdex 200 (GE healthcare) gel‐filtration column. The *in vitro* PDK1 kinase reaction was performed at room temperature in a final volume of 25 ml with purified PDK1 (30 nM) and PDKtide substrate peptide (8.4 μM, sequence: KTFCGTPEYLAPEVRREPRILSEEEQEMF ‐RDFDYIADWC) in a buffer containing 50 mM Tris (pH 7.5), 10 mM MgCl_2_, 50 mM ATP, 0.05 mg/mL bovine serum albumin, 0.005% Tween‐20, and 50 mM dithiothreitol, with or without indoprofen. The kinase activity was measured using an ADP‐Glo Kinase Assay kit (Promega), as per the supplied instructions.

### Library preparation and RNA sequencing

Sequencing libraries were prepared from total RNA (2 μg) using the TruSeq RNA Sample Prep Kit (Illumina) according to the manufacturer's instructions. Transcriptional profiles were analysed by e‐Biogen corporation. Libraries were prepared from total RNA using the SMARTer Stranded RNA‐Seq Kit (Clontech Laboratories). The isolation of mRNA was performed using the Poly(A) RNA Selection Kit (LEXOGEN). The isolated messenger (m)RNAs were used for the complementary DNA synthesis and shearing, following manufacture's instruction. Indexing was performed using the Illumina indexes 1–12. The enrichment step was carried out using of PCR. Subsequently, libraries were checked using the Agilent 2100 bioanalyser (DNA High Sensitivity Kit) to evaluate the mean fragment size. Quantification was performed using the library quantification kit using a StepOne Real‐Time PCR System (Life Technologies). High‐throughput sequencing was performed as paired‐end 100 sequencing using HiSeq 2500 (Illumina). For data analysis, mRNA‐Seq reads were mapped using tophat software^29^ tool in order to obtain the alignment file. Differentially expressed genes were determined based on counts from unique and multiple alignments using coverage in Bedtools.^30^ The Read Count data were processed based on quantile normalization method using edgeR within r 31 using Bioconductor. The alignment files also were used for assembling transcripts, estimating their abundances and detecting differential expression of genes or isoforms using cufflinks.^32^ And we used the fragments per kilobase of exon per million fragments as the method of determining the expression level of the gene regions.

The unbiased Gene Set Enrichment Analysis (GSEA; http://www.broadinstitute.org/gsea) was performed as described in previous studies.^33^, ^34^ All GSEA plots including GSEA enrichment plot and Enrichment Map were generated with the gsea software (Broad Institute; software.broadinstitute.org/gsea/). The Enrichment Map was exported with cytoscape (V3.7.2; https://cytoscape.org/index.html).

### *In vitro* and *in vivo* knockdown with short hairpin RNA

For the PDK1 short hairpin RNA lentivirus production, we used a lentivirus vector carrying a PDK1‐targeting short hairpin RNA (TRCN0000361331) (Sigma‐Aldrich) or the pLKO.1‐puro vector (negative control). HEK293T cells were transfected with these constructs using polyethylenimine (Polysciences, Inc.), and the transfected supernatants were harvested 2 days later. To test the knockdown efficiency, C2C12 cells were infected with lentiviruses in the presence of 8 μg/mL polybrene for 24 h, followed by the induction of differentiation in the presence of indoprofen or DMSO.

For *in vivo* lentiviral PDK1 knockdown, we concentrated the supernatants using PEG‐it™ Virus Precipitation Solution at 1,500 × *g* for 30 min at 4°C. The concentrated lentivirus pellets were resuspended in phosphate‐buffered saline. Briefly, mice were anesthetized with 5% isoflurane in 100% oxygen, and a 50 μL of lentivirus (9 × 10^8^ IU/mL) was injected into the tibialis anterior (TA) muscle three times, once every 3 days. The viral titre was determined using a colony formation assay.

### Statistical analysis

Values are expressed as mean ± standard deviation, as indicated in the figure legends. The statistical significance was calculated using Student's *t*‐test (paired, two‐tailed). Differences were considered statistically significant if *P* ≤ 0.05. For comparisons between multiple groups, the statistical significance was tested by analysis of variance test using spss v12.0 (SPSS, Chicago, IL).

## Results

### Indoprofen induces PGC‐1α expression in myoblasts and skeletal muscle of young mice

**Figure 1:**
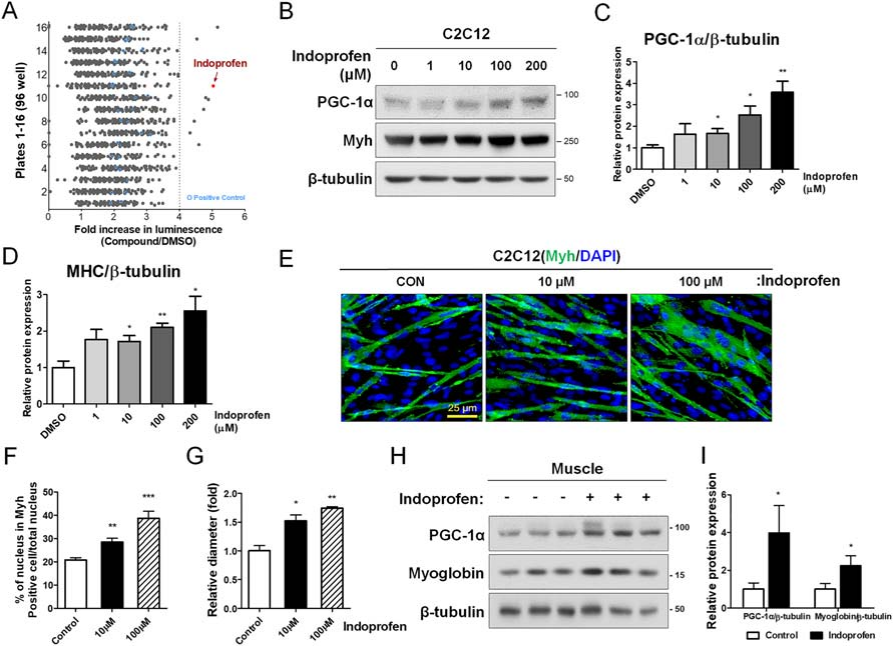
Effect of indoprofen on the expression of PGC‐1α in C2C12 and young mice skeletal muscle. (A) High‐throughput screening of 1280 small molecules at a single concentration (10 μM) in PGC‐1α‐Luc C2C12 cells. Each row represents a 96‐well plate. All read‐outs are displayed with colour‐coded shapes: grey circle (experimental compounds) and blue circle (AICAR, as a positive control). Arrow indicates data for indoprofen. (B) Western blot analysis for expression of PGC‐1α and Myh in C2C12 cells treated with the vehicle DMSO or 1, 10, 100, and 200 μM indoprofen for 2 days in differentiation medium. (C) Quantification of the relative levels of PGC‐1α proteins from panel A, *n* = 4. (D) Quantification of the relative levels of Myh proteins from panel A, *n* = 3. (E) Immunostaining for Myh expression in C2C12 cells treated with vehicle DMSO or 10, 100 μM indoprofen for 2 days. Scale bar, 25 μm. (F) Quantification of myotube formation in panel D. C2C12 cells were scored as percentage of nucleus in Myh positive cell/total nucleus per field, *n* = 4. (G) Quantification of Myh diameter per field, *n* = 3. (H) Western blot analysis for expression of PGC‐1α and myoglobin in gastrocnemius muscles from 3‐month‐old mice ingested with control or 2 mg/kg indoprofen for 4 weeks. (I) Quantification of the relative levels of PGC‐1α and myoglobin proteins from panel G, *n* = 3. Values are means of triplicates ±standard deviation. To determine statistical significance, an unpaired two‐tailed Student's *t*‐test was used (I), and a one‐way analysis of variance test with Tukey post‐hoc test was utilized (B–D, F, and G). Asterisk indicates statistical significance. ^*^ *P* < 0.05, ^**^ *P* < 0.01 and ^***^ *P* < 0.001 (Indoprofen vs. Control or DMSO). DMSO, dimethyl sulphoxide; MHC, myosin heavy chain; PGC‐1α, peroxisome proliferator‐activated receptor γ coactivator α.

To identify compounds that induce PGC‐1α expression, C2C12 myoblasts harbouring the luciferase gene under the control of a PGC‐1α promoter were used for a high‐throughput screen of Prestwick Chemical's 1280 Food and Drug Administration approved libraries. Primary read‐outs by luciferase assay were normalized to DMSO‐treated controls, and the top nine compounds that increased luciferase activity in the initial screen were selected for further study (*Figure* 1A). These compounds were then independently rescreened in triplicate. Among the selected PGC‐1α‐inducing candidates, we selected indoprofen, which exhibits the highest activity (*Figure* S1A). In a previous study, indoprofen was shown to enhance the survival motor neuron,^35^ which is critical for motor neuron function. Thus, indoprofen has the potential to exert synergistic effects to improve muscle function through enhancing motor input and muscle metabolism.

In agreement with the screening data, the treatment of indoprofen at increasing concentrations (1–200 μM) gradually elevated PGC‐1α protein levels, with a maximum increase of ~3.5‐fold, in C2C12 cells (*Figure* 1B and 1C). Further, the mRNA levels of PGC‐1α were also elevated in indoprofen‐treated in C2C12 cells compared with the control cells (*Figure* S1B). In addition, indoprofen treatment also elevated myosin heavy chain (Myh) levels and enhanced Myh‐positive myotube formation relative to DMSO‐treated C2C12 cells (*Figure* 1D–1G). To examine the specificity of indoprofen, two structurally similar drugs, indomethacin and ibuprofen, were also tested for their ability to inducible PGC‐1α in C2C12 cells. Unlike indoprofen, indomethacin and ibuprofen did not influence the expression of PGC‐1α or myoglobin, a protein with an important role in muscle oxidative metabolism, compared with the control (*Figure* S1C–S1E). Consistent with these findings, the myotube formation was also unaffected by these drugs (*Figure* S1F and S1G).

To assess the *in vivo* effects of indoprofen on PGC‐1α expression in skeletal muscle, 3‐month‐old mice were orally administered vehicle or indoprofen (2 mg/kg) for 4 weeks, and their muscles were analysed by immunoblotting. The levels of PGC‐1α and myoglobin were substantially enhanced in indoprofen‐treated muscles (*Figure* 1H and 1I). These data demonstrate that indoprofen increases PGC‐1α levels in myoblasts and muscles.

### Indoprofen enhances oxidative muscle metabolism

**Figure 2:**
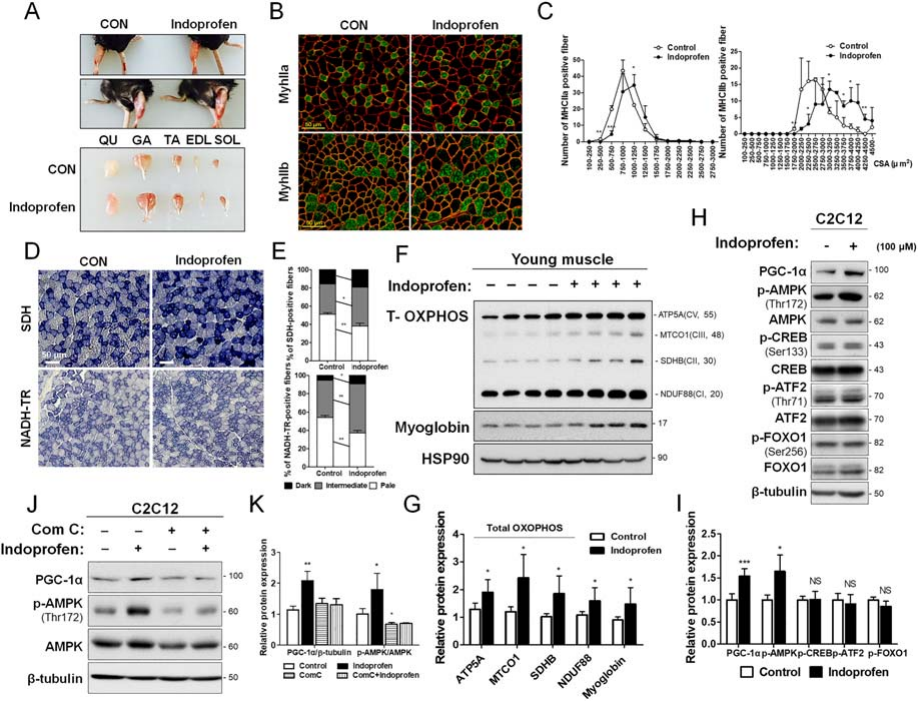
Effect of indoprofen on the oxidative muscle metabolism and PGC‐1α regulatory targets. (A) Photographs of isolated muscle types from hindlimbs from 3‐month‐old mice ingested with control or 2 mg/kg indoprofen for 4 weeks. (B) Immunostaining of MyhIIa (green), MyhIIb (green), and laminin (red) in the TA muscles of control or 2 mg/kg indoprofen‐ingested mice for 4 weeks. Scale bar, 50 μm. (C) Quantification of MyhIIa‐(Control: 263, Indoprofen: 262) and MyhIIb‐(Control: 160, Indoprofen: 169) positive myofibres in panel B, *n* = 3. (D) Histochemical staining for SDH and NADH‐TR enzymatic activities in EDL muscles. Scale bar, 50 μm. (E) The staining intensities of SDH (upper) and NADH‐TR (lower) are quantified as three different grades (dark, intermediate, and pale) and plotted as a percentile, *n* = 3. (F) Western blot analysis for expression of total‐OXPHOS and Myoglobin in quadriceps muscles from 3‐month‐old mice ingested with control or 2 mg/kg indoprofen for 4 weeks. (G) Quantification of the relative levels of total OXPHOS and myoglobin proteins from panel F, *n* = 4. (H) Western blot analysis for expression of PGC‐1α, p‐AMPK, AMPK, p‐CREB, CREB, p‐ATF2, ATF2, p‐FOXO1, and FOXO1 of C2C12 cells treated with the vehicle DMSO or indoprofen (100 μM) for 24 h in differentiation medium. (I) Quantification of the relative levels of proteins from panel H, *n* = 3. (J) Western blot analysis for expression of PGC‐1α, p‐AMPK, and AMPK of C2C12 cells treated with vehicle, indoprofen (100 μM), or Compound C (2 μM) alone or in the combination for 24 h in differentiation medium. (K) Quantification of the relative levels of PGC‐1α proteins from panel J, *n* = 3. For the calculation of relative phosphorylation levels, the densitometries of the immunoblots of the phospho‐Abs were normalized to the total protein levels. Data are expressed as mean ± standard deviation. To determine statistical significance, an unpaired two‐tailed Student's *t*‐test was used (C, E, G, and I) and two‐way analysis of variance test with Tukey post‐hoc test was utilized (K). ^*^ *P* < 0.05, ^**^ *P* < 0.01, and ^***^ *P* < 0.001 (Indoprofen vs. Control). AMPK, AMP‐activated protein kinase; ATF2, activating transcription factor‐2; CREB, cAMP response element‐binding protein; DMSO, dimethyl sulphoxide; EDL, extensor digitorum longus; GA, gastrocnemius; NADH‐TR, nicotinamide adenine dinucleotide‐tetrazolium reductase; MHC, myosin heavy chain; PGC‐1α, peroxisome proliferator‐activated receptor γ coactivator α; QU, quadriceps; SDH, succinate dehydrogenase; SOL, soleus; TA, tibialis anterior.

The functional consequences of elevated PGC‐1α levels following indoprofen treatment were assessed in 3‐month‐old mice treated with control or indoprofen for 4 weeks. The hindlimb muscles of the indoprofen‐treated mice appeared darker than those of control mice, which could be readily detected in gastrocnemius muscles among the five hindlimb muscles, including the quadriceps, TA, extensor EDL, and SOL (*Figure* 2A). Indoprofen treatment increased the proportion of larger myofibres (*Figure* S2D and S2E) and significantly enhanced the proportion of larger MyhIIa‐positive and MyhIIb‐positive myofibres compared with the control treatment (Figures 2B, 2C, S2F, and S2G). In addition, muscles were subjected to histochemical staining for activities of two mitochondrial enzymes, SDH and nicotinamide adenine dinucleotide‐tetrazolium reductase (NADH‐TR). Indoprofen treatment elevated the proportion of myofibres with strong (dark) and intermediate activities for SDH and NADH‐TR compared with control muscles (*Figure* 2D and 2E). In addition, indoprofen enhanced total‐OXPHOS [ATP5A (CV), MTCO1 (CIII), SDHB (CII) and NDUF88 (CI)] and myoglobin expression (*Figure* 2F and 2G).

Peroxisome proliferator‐activated receptor γ coactivator α transcription and activity are controlled by a range of upstream signalling molecules and pathways, including AMPK and p38 mitogen‐activated protein kinase.^36^, ^37^, ^38^ Additionally, CREB, ATF2, and FOXO1 transcription factors have been implicated in PGC‐1α induction.^39^, ^40^, ^41^ The indoprofen treatment of C2C12 cells for 24 h elevated levels of PGC‐1α levels and p‐AMPK while the levels of phosphorylated or total CREB, ATF2, and FOXO1 were unchanged, compared with the DMSO‐treated C2C12 cells (*Figure* 2H and 2I). To further assess whether the effects of indoprofen on PGC‐1α induction are dependent on AMPK, C2C12 cells were treated with DMSO or indoprofen along with an AMPK inhibitor, Compound C. As expected, Compound C treatment attenuated the indoprofen effects on AMPK activation and PGC‐1α induction (*Figure* 2J and 2K). Taken together, these results suggest that indoprofen treatment induces PGC‐1α by AMPK activation, thereby contributing to enhanced oxidative muscle metabolism.

### Indoprofen enhances muscle mass and anabolic signalling in young mice

**Figure 3:**
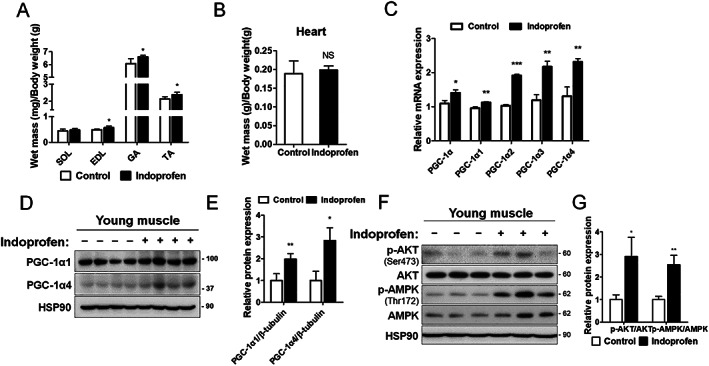
Effect of indoprofen on the upstream pathway of PGC‐1α for muscle mass increase. (A) Weights of four muscle types from control or 2 mg/kg indoprofen ingested mice for 4 weeks, *n* = 4. (B) The heart muscle mass of control and 2 mg/kg indoprofen treated mice 4 weeks, *n* = 4. (C) qRT‐PCR analysis for expression of PGC‐1α isoforms in gastrocnemius muscles from control or 2 mg/kg indoprofen‐ingested mice 4 weeks, *n* = 4. (D) Western blot analysis for expression of PGC‐1α1 and PGC‐1α4 in quadriceps muscles from 3‐month‐old mice ingested with control or 2 mg/kg indoprofen for 4 weeks. (E) Quantification of the relative levels of PGC‐1α1 and PGC‐1α4 proteins from panel D, *n* = 4. (F) Western blot analysis for expression of p‐AKT, AKT, p‐AMPK, and AMPK in quadriceps muscles from 4‐month‐old mice ingested with control or 2 mg/kg indoprofen for 4 weeks. (G) Quantification of the relative levels of proteins from panel F, *n* = 3. For the calculation of relative phosphorylation levels, the densitometries of the immunoblots of the phospho‐Abs were normalized to the total protein levels. Data are expressed as mean ± standard deviation. To determine statistical significance, the Student's *t*‐test was used. ^*^ *P* < 0.05, ^**^ *P* < 0.01 and ^***^ *P* < 0.001 (Indoprofen vs. Control). AMPK, AMP‐activated protein kinase; ATF2, activating transcription factor‐2; CREB, cAMP response element‐binding protein; DMSO, dimethyl sulphoxide; EDL, extensor digitorum longus; GA, gastrocnemius; NADH‐TR, nicotinamide adenine dinucleotide‐tetrazolium reductase; MHC, myosin heavy chain; PGC‐1α, peroxisome proliferator‐activated receptor γ coactivator α; SDH, succinate dehydrogenase; SOL, soleus; TA, tibialis anterior.

To evaluate the effects of indoprofen on muscle mass, young mice were treated with vehicle or indoprofen for 4 weeks, and muscles were harvested 4 h after the last administration. Among the four hindlimb muscles, the weights of gastrocnemius, EDL, and TA muscles were significantly increased by indoprofen treatment (*Figure* 3A). Consistent with this finding, the protein synthesis and the level of total protein were also elevated in indoprofen‐treated C2C12 cells compared with the control (*Figure* S1H–JS1). However, heart mass was unaffected (*Figure* 3B), and treatment with 2 mg/kg indoprofen did not cause changes in body weight, food intake, and water intake of mice (*Figure* S2A–S2C). These data suggest that indoprofen has a boosting effect in skeletal muscle without exerting overt hypertrophic effects on cardiac muscle or inducing severe effects in the whole body. The qRT‐PCR analysis revealed that all forms of PGC‐1α, including the PGC‐1α4 isoform that is linked with skeletal muscle hypertrophy,^42^ were significantly increased by indoprofen treatment (*Figure* 3C). Further, the protein levels of PGC‐1α1 and PGC‐1α4 were also elevated in indoprofen‐treated muscles compared with the control muscles (*Figure* 3D and 3E). Next, we examined the effects of indoprofen on the activation of AKT, a key anabolic mediator. Indoprofen‐treated muscles had substantially enhanced p‐AKT levels, in addition to increase p‐AMPK levels, compared with control muscles (*Figure* 3F and 3G). Taken together, these results suggest that indoprofen treatment leads to increased muscle mass and augments AMPK and AKT activation in muscles.

### Indoprofen improves muscle function and mass in aged mice

**Figure 4:**
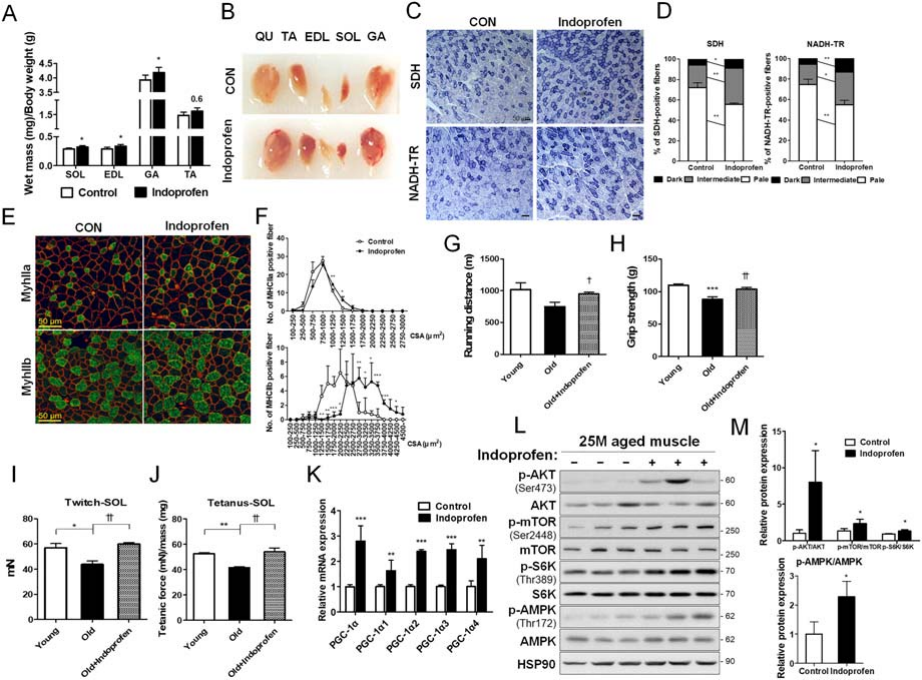
Effect of indoprofen on skeletal muscle function and mass in old mice. (A) Weights of four muscle types from control or 2 mg/kg indoprofen ingested 25‐month‐old mice, *n* = 6. (B) Photographs of isolated muscle types from hindlimbs from 25‐month‐old mice ingested with control or indoprofen for 12 weeks. (C) Histochemical staining for SDH and NADH‐TR enzymatic activities in EDL muscles. Scale bar, 50 μm. (D) The staining intensities of SDH (left) and NADH‐TR (right) are quantified as three different grades (dark, intermediate, and pale) and plotted as a percentile, *n* = 3. (E) Immunostaining of MyhIIa (green), MyhIIb (green) and laminin (red) in the TA muscles of control or 2 mg/kg indoprofen‐ingested 25‐month‐old mice for 12 weeks. Scale bar, 50 μm. (F) Quantification of MyhIIa‐(Control: 136, Indoprofen: 119) and MyhIIb‐(Control: 100, Indoprofen: 102) positive myofibres in panel E, *n* = 3. (G) The endurance activity on treadmill is depicted a distance in young‐control (4‐month‐old), old‐control (25‐month‐old), or old‐indoprofen (2 mg/kg) ingested mice for 12 weeks, *n* = 4. (H) Grip strength depicted as the force (gram) that animals in each group pulled, *n* = 6. (I) Isometric twitch contractions measurement for soleus muscle function in young‐control (7‐month‐old), old‐control (25‐month‐old), or old‐indoprofen (2 mg/kg) ingested mice for 12 weeks, *n* = 4. (J) Isometric tetanic contractions measurement for isolated soleus muscle function, *n* = 4. Force was normalized to the muscle mass (mg). (K) qRT‐PCR analysis for expression of PGC‐1α isoforms in muscles of control or 2 mg/kg indoprofen‐ingested 25‐month‐old mice for 12 weeks, *n* = 4. (L) Western blot analysis for expression of p‐AKT, AKT, p‐mTOR, mTOR, p‐S6K, S6K, p‐AMPK, and AMPK in TA muscles from 25‐month‐old mice ingested with control or 2 mg/kg indoprofen for 12 weeks. (M) Quantification of the relative levels of proteins from panel L, *n* = 3. For the calculation of relative phosphorylation levels, the densitometries of the immunoblots of the phospho‐Abs were normalized to the total protein levels. Data are expressed as mean ± standard deviation. To determine statistical significance, the Student's *t*‐test was used (A, D, F, K, and M) and a two‐way analysis of variance test with Tukey post‐hoc test was utilized (G–J). ^*^ *P* < 0.05, ^**^ *P* < 0.01, and ^***^ *P* < 0.001 (Indoprofen vs. Control); ^†^ *P* < 0.05 and ^††^ *P* < 0.01 (Old+Infoprofen vs. Old). AMPK, AMP‐activated protein kinase; ATF2, activating transcription factor‐2; CREB, cAMP response element‐binding protein; DMSO, dimethyl sulphoxide; EDL, extensor digitorum longus; GA, gastrocnemius; NADH‐TR, nicotinamide adenine dinucleotide‐tetrazolium reductase; MHC, myosin heavy chain; PGC‐1α, peroxisome proliferator‐activated receptor γ coactivator α; SDH, succinate dehydrogenase; SOL, soleus; TA, tibialis anterior.

To determine the effects of indoprofen on muscle function and mass in aged mice, 22‐month‐old mice were treated with vehicle or indoprofen for 3 months. The experimental conditions used in this study did not cause any punctate lesions or gastrointestinal bleeding at 2 mg/kg indoprofen treatment (*Figure* S3A). Unlike 800 mg indoprofen per day in humans, which is known to cause severe side effects,^43^ the low concentration (corresponding to 100 mg in humans) used in our animal experiment (2 mg/kg in mice) did not cause gastrointestinal bleeding or changes in body weight (*Figure* S3A and S3B). Similar to the effect observed in young mice, there was a significant boosting effect of indoprofen on the weights of four hindlimb muscles in aged mice, without overt hypertrophic effect on cardiac muscle, compared with control aged mice (*Figure* 4A and S3C). In addition, the hindlimb muscles of indoprofen‐treated mice appeared darker than those of control aged mice (*Figure* 4B). TA muscles from indoprofen‐treated aged mice had an increased proportion of myofibres with strong and intermediate activities for SDH and NADH‐TR relative to muscles from control aged mice (*Figure* 4C and 4D). In addition, indoprofen treatment significantly enhanced total‐OXPHOS expression in aged muscles (*Figure* S3D and S3E). An examination of myofibre size and types revealed that indoprofen treatment increased the proportion of larger MyhIIa‐positive and MyhIIb‐positive myofibers (*Figure*s S3F–I, 4E, and 4F).

The effects of indoprofen on mouse muscle function were assessed by measuring running distance, grip strength, and isometric force. Although control aged mice exhibited an approximately 20% reduction in muscle strength relative to control young mice, indoprofen treatment of aged mice restored muscle function almost to the level of control young mice (*Figure* 4G–4J). Similar to the results obtained with young mice, the expression levels of PGC‐1α isoforms were greatly increased with indoprofen treatment in aged muscles (*Figure* 4K). In addition, indoprofen treatment enhanced the level of p‐AMPK and the anabolic signalling components, p‐AKT, p‐mTOR, and p‐S6K (*Figure* 4L and 4M). These data collectively suggest that the indoprofen treatment improves muscle function and mass in aged mice through activation of both the AMPK and AKT/S6K pathways.

### Indoprofen reverses the expression of aging‐associated genes

**Figure 5:**
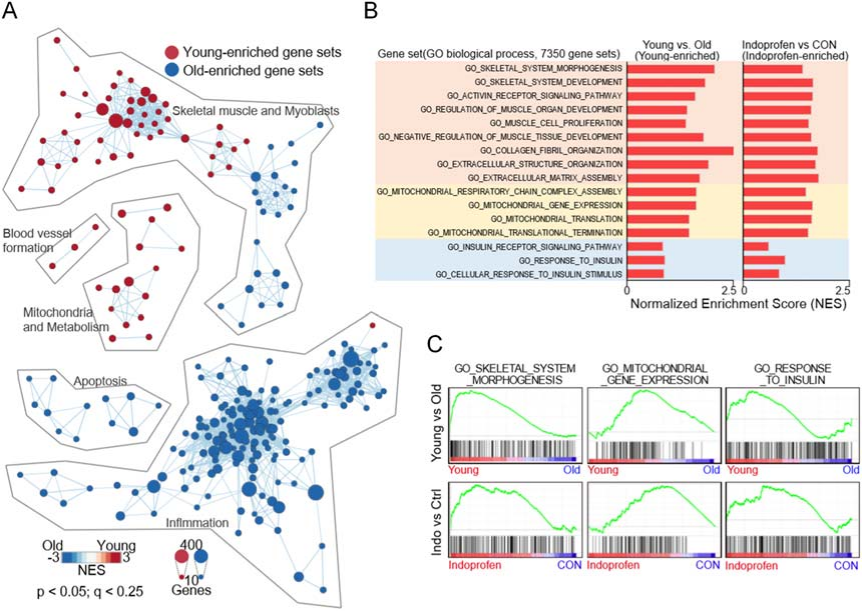
Gene expression profiles in the muscles of indoprofen‐fed aged mice. (A) The enrichment map summarizing the result of Gene Set Enrichment Analysis of the young and old skeletal muscle transcriptomes. (B) The bar charts showing the normalized enrichment score (NES) of the selected gene sets such as skeletal muscle and myoblast‐related, mitochondria‐related, and insulin receptor signalling pathway‐related gene set (Left, young muscle‐enriched gene sets; right, indoprofen‐administrated muscle‐enriched gene sets). (C) The selected Gene Set Enrichment Analysis plots presenting the similar enrichment patterns in young and 2 mg/kg indoprofen‐administrated old mice muscles for 12 weeks.

To understand the boosting effect of indoprofen on aged muscles, we performed unbiased gene set enrichment analysis using the skeletal muscle transcriptomes of young or old mice, and with vehicle or indoprofen treatment of mice. Among 7350 gene sets categorized to gene ontology biological process, the expression patterns of 566 gene sets were altered with statistical significance (*Figure* 5A). To see age‐dependent functional enrichment, those gene sets were visualized with the enrichment map visualization method. The red or blue nodes indicate the young‐enriched or old‐enriched gene sets, respectively. The size of a node indicates the size of each gene set, and the edge means two connected nodes share some genes. Skeletal muscle‐related and mitochondria‐related gene sets were enriched in the young muscle (reduced in the aged muscle). The reduced expression of those genes in aged muscles links to mitochondrial dysfunction during muscle aging, as reported previously.^44^ Remarkably, the young muscle‐enriched gene sets were also enriched in the muscles of indoprofen‐treated old mice (*Figure* 5B, 5C, and S4A), suggesting that indoprofen reverses the muscle transcriptomic signature toward a young signature.

### Indoprofen prevents muscle weakness in muscle atrophy model

**Figure 6:**
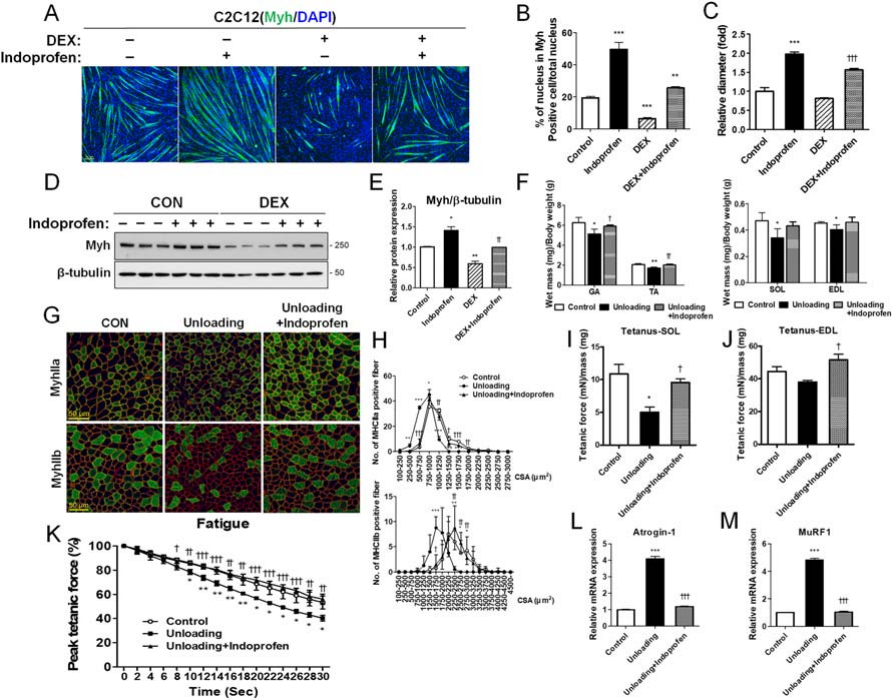
Effect of indoprofen on destruction of muscle atrophy model. (A) Immunostaining for Myh expression in C2C12 cells treated with normal differentiation medium for 1 day and treated with vehicle, indoprofen (100 μM), or dexamethasone (10 μM) alone or in the combination for 2 days in differentiation medium. Scale bar, 100 μm. (B) Quantification of myotube formation in panel A. C2C12 cells were scored as percentage of nucleus in Myh positive cell/total nucleus per field, *n* = 4. (C) Quantification of Myh‐positive myotube diameter per field, *n* = 3. (D) Western blot analysis of Myh expression in C2C12 cells normal differentiation medium for 1 day and treated with vehicle, indoprofen (100 μM), or dexamethasone (10 μM) alone or in the combination for 2 days. (E) Quantification of the relative levels of Myh proteins from panel D, *n* = 3. (F) Weights of four muscle types from control, unloading (for 2 weeks)‐control or unloading‐indoprofen (2 mg/kg) ingested 3‐month‐old mice, *n* = 4. (G) Immunostaining of TA muscles for expression of MyhIla and MyhIIb from control, unloading‐control or unloading‐indoprofen ingested 3‐month‐old mice, Scale bar, 50 μm. (H) Quantification of MyhIIa‐(Control: 154, Unloading: 189, Unloading+Indoprofen: 197) and MyhIIb‐(Control: 116, Unloading: 104, Unloading+ Indoprofen: 125) positive myofibers in panel G, *n* = 3. (I) Isometric tetanic contractions measurement for isolated SOL muscle function, *n* = 3. Force was normalized to the muscle mass (mg). (J) Isometric tetanic contractions measurement for isolated EDL muscle function, *n* = 3. Force was normalized to the muscle mass (mg). (K) Fatigue‐resistance assay of the SOL muscle. Fatigue was recorded by the decline of relative force to the initial force by repetitive stimulations at a given time, *n* = 3. (L) qRT‐PCR analysis for expression of Atrogin‐1 in quadriceps muscles from control, unloading‐control, or unloading‐indoprofen‐ingested mice, *n* = 4. (M) qRT‐PCR analysis for expression of MuRF1 in quadriceps muscles from control, unloading‐control, or unloading‐indoprofen‐ingested mice, *n* = 4. Data are expressed as mean ± standard deviation. To determine statistical significance, analysis of variance test with Tukey post‐hoc analysis (one‐way, F; two‐way, B, C, E, and I–M) was utilized. ^*^ *P* < 0.05, ^**^ *P* < 0.01 and ^***^ *P* < 0.001 (Indoprofen vs. Control); ^†^ *P* < 0.05, ^††^ *P* < 0.01, and ^†††^ *P* < 0.001 (DEX + Infoprofen vs. DEX and Unloading+Indoprofen vs. Unloading). AMPK, AMP‐activated protein kinase; ATF2, activating transcription factor‐2; CREB, cAMP response element‐binding protein; DEX, dexamethasone; DMSO, dimethyl sulphoxide; EDL, extensor digitorum longus; GA, gastrocnemius; NADH‐TR, nicotinamide adenine dinucleotide‐tetrazolium reductase; MHC, myosin heavy chain; SOL, soleus; TA, tibialis anterior.

Muscle atrophy can be triggered by treatment with dexamethasone (DEX) or muscle disuse, which accelerate muscle protein degradation through induction of muscle specific ubiquitin ligases MAFbx/Atrogin‐1 and MuRF1.^45^ We next examined the effects of indoprofen on the DEX‐mediated myotube atrophy. C2C12 cells were co‐treated with DEX in combination with vehicle or indoprofen for 2 days. Treatment with DEX alone starkly reduced myotube formation, in both number and size, relative to vehicle DMSO treatment (*Figure* 6A–6C). Co‐treatment of DEX and indoprofen blocked the reduction of myotube formation. Consistent with this result, DEX treatment significantly reduced Myh protein levels, which were partially restored by indoprofen treatment (*Figure* 6D and 6E). Furthermore, the expression levels of p‐AKT and Atrogin‐1 were also greatly changed in DEX‐treated C2C12 cells, while indoprofen treatment abrogated these changes (*Figure* S4B and S4C).

Next, to further evaluate the inhibitory effect of indoprofen treatment on muscle protein degradation likely contributing to muscle mass increase, the effects of indoprofen on disuse‐triggered muscle atrophy were assessed. Muscle atrophy was triggered by hindlimb suspension unloading for 2 weeks. The weights of hindlimb muscles from the atrophy model were reduced by about 10–30% compared with the control mice (*Figure* 6F). Indoprofen treatment significantly prevented the disuse‐triggered muscle loss compared with the control. In addition, indoprofen treatment prevented the decrease of myofibre size induced by disuse (*Figures* 6G, 6H, and S4D–H). The muscle strength measured by the isometric contraction of SOL and EDL muscles revealed that indoprofen treatment protected muscles from contractile force decline in disuse mice (*Figures* 6I, 6J, and S4I). During electrical muscle activation, fatigue was aggravated in disuse muscles, whereas muscles from the control or the indoprofen‐treated disuse mice exhibited fatigue‐resistant properties (*Figure* 6K). Consistent with this phenotype, mRNA expression levels of Atrogin‐1 and MuRF1 were highly upregulated in disuse muscles, while the indoprofen treatment abrogated this induction (*Figure* 6L and 6M). Taken together, these data suggest that indoprofen treatment averts muscle atrophy triggered by disuse through repression of muscle specific ubiquitin ligases.

### 3‐Phosphoinositide‐dependent protein kinase‐1 promotes AKT/p70S6 kinase activation in response to indoprofen

To further define the mechanisms of indoprofen effects, the reporter activities mediated by full‐length or mutant PGC‐1α promoters (with a deletion of the MEF2 or CRE sites) were measured (*Figure* S5A–S5C). In agreement with our previous data, the full‐length promoter‐driven luciferase activities were significantly upregulated by indoprofen, while deletion of the MEF2 or CRE sites abrogated the responsiveness to indoprofen. Considering that AKT can suppress the expression of Atrogin‐1 and MuRF1^17^, ^18^ and induce the transcription mediated by MEF2C or CREB through MEF2 or CRE sites, respectively^46^, ^47^; the preventive effects of indoprofen against muscle loss can be attributed to meditation of the AKT‐mediated anabolic pathway.

**Figure 7:**
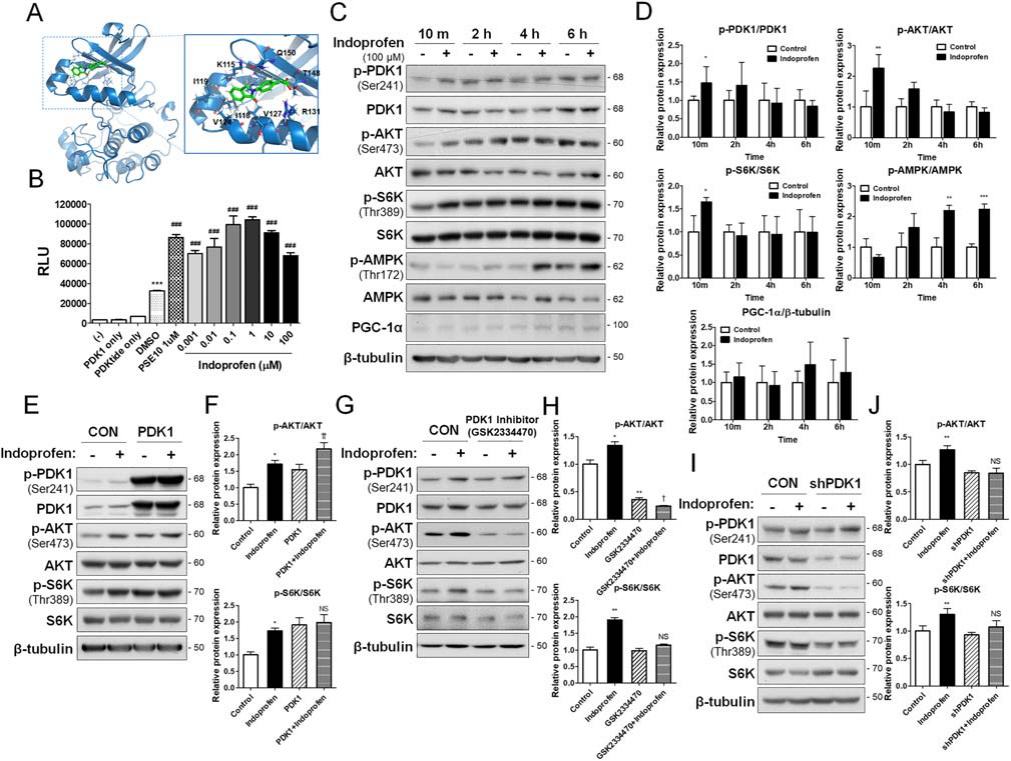
The effect of indoprofen is mediated by PDK1 and downstream anabolic pathways. (A) A representation of the computationally predicted cluster of indoprofen bound to PDK1 (Protein Data Bank code 4AW1). Predictions place indoprofen (green) in the PIF‐pocket site. (B) PDK1 activity was measured in the presence and absence of 1 μM PSE10 (as a positive control) and various concentration (0.001~100 μM) of indoporfen using *in vitro* protein kinase assay, *n* = 3. (C) Western blot analysis for expression of p‐PDK1, PDK1, p‐AKT, AKT, p‐S6K, S6K, p‐AMPK, AMPK and PGC‐1α of C2C12 cells treated with vehicle or indoprofen (100 μM) for 10 minutes, 2 hours, 4 hours, and 6 hours in stabilized differentiation medium condition. (D) Quantification of the relative levels of proteins from panel B, *n* = 3. (E) Western blot analysis for expression of p‐PDK1, PDK1, p‐AKT, AKT, p‐S6K and S6K of C2C12 cells transfected PDK1 overexpression treated with vehicle or indoprofen (100 μM) for 10 minutes in stabilized differentiation medium condition. (F) Quantification of the relative levels of p‐PDK1, p‐AKT and p‐S6K proteins from panel E, *n* = 3. (G) Western blot analysis for expression of p‐PDK1, PDK1, p‐AKT, AKT, p‐S6K and S6K of C2C12 cells treated with vehicle or indoprofen (100 μM) with or without GSK2334470 (3 μM) for 4 hours in stabilized differentiation medium condition. (H) Quantification of the relative levels of p‐AKT and p‐S6K proteins from panel G, *n* = 3. (I) Western blot analysis for p‐PDK1, PDK1, p‐AKT, AKT, p‐S6K and S6K of C2C12 cells transfected lentiviral PDK1 knockdown treated with vehicle or indoprofen (100 μM) for 10 minutes in stabilized differentiation medium condition. (J) Quantification of the relative levels of p‐AKT and p‐S6K proteins from panel I, *n* = 3. For the calculation of relative phosphorylation levels, the densitometries of the immunoblots of the phospho‐Abs were normalized to the total protein levels. Data are expressed as mean ± SD. To determine statistical significance, an unpaired two‐tailed student t‐test was used (D) and ANOVA test with Tukey post‐hoc analysis (one‐way, B; two‐way, F, H, and J) was utilized. *p < 0.05, **p < 0.01, and ***p < 0.001 (Indoprofen, PDK1 or PDK1 + Indoprofen vs. Control). AMPK, AMP‐activated protein kinase; ATF2, activating transcription factor‐2; CREB, cAMP response element‐binding protein; DMSO, dimethyl sulphoxide; EDL, extensor digitorum longus; GA, gastrocnemius; NADH‐TR, nicotinamide adenine dinucleotide‐tetrazolium reductase; MHC, myosin heavy chain; PGC‐1α, peroxisome proliferator‐activated receptor γ coactivator α; PDK1, phosphoinositide‐dependent protein kinase‐1; SDH, succinate dehydrogenase; SOL, soleus; TA, tibialis anterior.

To identify indoprofen's potential target(s), we applied the drug to the ligand‐based *in silico* target search server, PharmMapper (http://lilab.ecust.edu.cn/pharmmapper/). As a top ranked candidate, PDK1, an upstream kinase of AKT, was identified (*Table* S2). Using BIOVIA Discovery Studio, structural modelling of the indoprofen‐PDK1 interaction was calculated and the CDOCKER interaction energy between indoprofen and PDK1 was estimated at −50.452 kcal/mol (*Figure* 7A). Several publications reporting drug–protein interactions indicate that compounds with about −50 kcal/mol of a CDOCKER Interaction energy would work as effector molecules to their putative targets.^48^, ^49^, ^50^ In optimized reaction conditions (10 min kinase reaction time, *Figure* S6A and S6B), *in vitro* protein kinase assays demonstrated that indoprofen activated the biochemical activity of PDK1 (*Figure* 7B). BIAcore analysis also showed that indoprofen binds PDK1 *in vitro* (*Figure* S6C). Considering that PDK1 acts as an upstream kinase of AKT‐mediated anabolic pathway with a previously proposed role in determining cell size,^51^ we hypothesized that PDK1 might mediate the indoprofen effect on muscle mass. To test this hypothesis, C2C12 cells were treated with DMSO or indoprofen for a short time (10 min) or longer periods (2–6 h), followed by immunoblotting for signalling proteins (*Figure* 7C and 7D). The short indoprofen treatment enhanced the level of the active and phosphorylated form of PDK1, AKT, and S6K compared with the control treatment, while the level of p‐AMPK was unchanged following 10 min of indoprofen treatment. Interestingly, the levels of active PDK1, AKT, and S6K did not change following indoprofen treatment for 2, 4, or 6 h, while p‐AMPK levels were greatly elevated and the expression level of PGC‐1α was also slightly increased in response to these longer indoprofen treatments. In addition, to further assess whether indoprofen specifically regulates PDK1 and downstream signalling, the levels of signalling proteins upstream of PDK1s were measured. The levels of the active phosphorylated forms of IRS and PI3K and tyrosine kinase signalling using a phospho‐tyrosine antibody were unchanged by indoprofen treatment for all treatment periods in C2C12 cells (*Figure* S6E and S6F). These data suggest that indoprofen induces a sequential activation of anabolic and catabolic kinases, resembling the molecular responses following resistance exercise.^23^

To further investigate the role of PDK1 in the indoprofen effect on muscle mass, control and PDK1‐overexpressing C2C12 cells were treated with indoprofen for 10 min. PDK1 overexpression alone enhanced p‐AKT and p‐S6K levels, similarly to the indoprofen treatment of control cells (*Figure* 7E and 7F). The indoprofen treatment further increased p‐AKT levels induced by PDK1 overexpression, while p‐S6K levels were not further increased. Conversely, the inhibition of PDK1 with a PDK1 inhibitor, GSK2334470, greatly attenuated AKT and S6K activation in control and indoprofen‐treated cells (*Figure* 7G and 7H). Furthermore, the depletion of PDK1 by shPDK1‐expressing lentiviral infection abrogated the upregulation of p‐PDK1, p‐AKT, p‐S6K, PGC‐1α, and mitochondria‐related gene levels in response to indoprofen (*Figures* 7I, 7J, S6G, and S6H). Moreover, to determine whether indoprofen regulates PGC‐1α expression through AKT and/or AMPK, the reporter activities mediated by the full‐length or mutant PGC‐1α promoters (with a deletion of the MEF2 or CRE site) were measured in PDK1‐inhibited and Compound C‐treated cells. In agreement with our previous data, the full‐length promoter‐driven luciferase activities were significantly upregulated by indoprofen, while the deletion of the MEF2 or CRE sites abrogated the responsiveness to indoprofen. In addition, the depletion of PDK1 and the treatment of Compound C also attenuated the indoprofen effect on PGC‐1α induction (*Figure* S5D and S5E). Collectively, these data suggest that the indoprofen effect on AKT and S6K activation is mediated by PDK1.

### 3‐Phosphoinositide‐dependent protein kinase‐1 is required for the preventive effects of indoprofen on muscle atrophy

**Figure 8:**
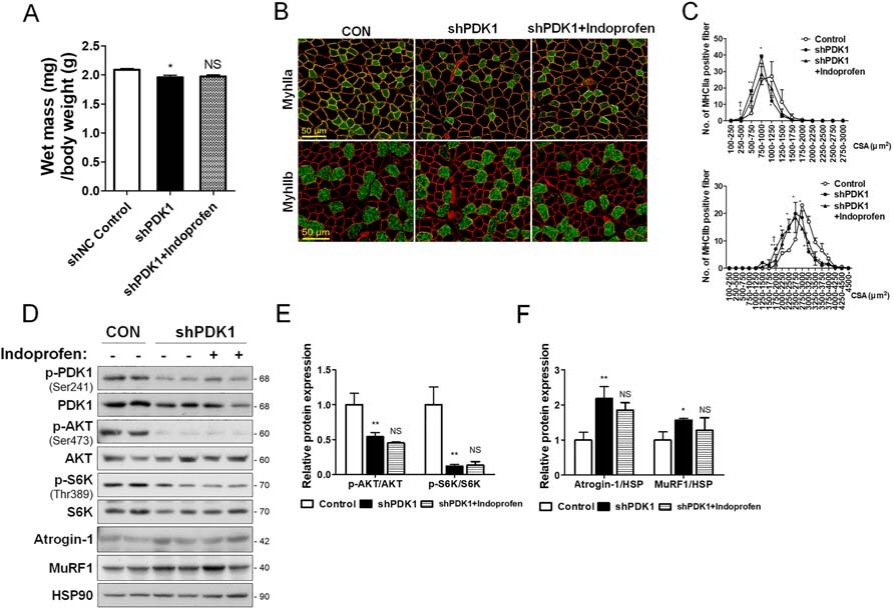
Effect of indoprofen on PDK1 knockdown *in vivo* mice model. (A) Weights of four muscle types from lentiviral shNC‐treated control, shPDK1‐treated control and shPDK1‐treated 2 mg/kg indoprofen ingested 3‐month‐old mice for 2 weeks, *n* = 4. (B) Immunostaining of tibialis anterior muscles for expression of MyhIIa and MyhIIb from lentiviral shNC‐treated control, shPDK1‐treated control and shPDK1‐treated 2 mg/kg indoprofen ingested 3‐month‐old mice for 2 weeks. Scale bar, 50 μm. (C) Quantification of MyhIIa‐(Control: 142, shPDK1: 143, shPDK1 + Indoprofen: 125) and MyhIIb‐(Control: 169, shPDK1: 162, shPDK1 + Indoprofen: 159) positive myofibers in panel B. *n* = 3. (D) Western blot analysis for expression of p‐PDK1, PDK1, p‐AKT, AKT, p‐S6K, S6K, Atrogin‐1, and MuRF1 in tibialis anterior muscles from lentiviral shNC‐treated control, shPDK1‐treated control and shPDK1‐treated 2 mg/kg indoprofen ingested 3‐month‐old mice for 2 weeks. (E) Quantification of the relative levels of p‐AKT and p‐S6K proteins from panel D, *n* = 3. (F) Quantification of the relative levels of Atrogin‐1 and MuRF1 proteins from panel D, *n* = 3. For the calculation of relative phosphorylation levels, the densitometries of the immunoblots of the phospho‐Abs were normalized to the total protein levels. Data are expressed as mean ± standard deviation. To determine statistical significance, two‐way analysis of variance test with Tukey post‐hoc test was utilized. ^*^ *P* < 0.05, ^**^ *P* < 0.01, and ^***^ *P* < 0.001 (shPDK1 vs. Control or shNC Control); NS (not significant), ^†^ *P* < 0.05, and ^††^ *P* < 0.01 (shPDK1 + Indoprofen vs. shPDK1). MHC, myosin heavy chain; PDK1, phosphoinositide‐dependent protein kinase‐1; S6k, AKT/p70S6 kinase.

To corroborate the role of PDK1 mediating the muscle boosting effect of indoprofen, TA muscles of mice pretreated with vehicle or indoprofen for 1 week were injected with control or shPDK1 lentiviruses and treated for an additional 2 weeks. The shPDK1 lentivirus‐infected TA muscles exhibited a mild but significant muscle loss, which was unchanged by indoprofen treatment, compared with the control muscles (*Figure* 8A). The immunostaining of muscles with for laminin, MyhIIa, or MyhIIb revealed that the cross‐sectional area of myofibres was decreased in PDK1‐depleted muscles and indoprofen treatment did not influence this reduction, relative to the control virus‐transduced muscles (*Figures* 8B and 8C and S6I–K). Consistent with this finding, AKT and S6K activation were greatly decreased by PDK1 depletion, and these effects were also unaltered by indoprofen treatment (*Figure* 8D and 8E). In addition, the levels of Atrogin‐1 and MuRF1 were increased by PDK1 inhibition, and these effects were unaffected by indoprofen treatment (*Figure* 8D and 8F). These data suggest that the effects of indoprofen on activation of anabolic pathway and suppression of Atrogin‐1 and MuRF1 are mediated by PDK1. In summary, indoprofen represents an effective drug to prevent muscle loss and weakness associated with aging or pathological conditions.

## Discussion

In the current study, we characterized the effects of indoprofen, which we had identified as an inducer of PGC‐1α using high‐throughput screening. Consistent with the initial screening data, indoprofen enhanced PGC‐1α expression, accompanied by an increase in mitochondrial OXPHOS proteins and oxidative enzyme activity. These results are consistent with previous studies showing that the enhanced expression of PGC‐1α has been linked to the prevention of muscle weakness and aging.^3^, ^7^, ^8^ Notably, indoprofen treatment improved the muscle mass and function in young and aged mice and prevented the disuse‐triggered muscle wasting accompanied by induction of MuRF1 and Atrogin‐1 expression. Indoprofen seems to exert these effects through PDK1, which in turn activates the AKT/S6K pathway.

PDK1 plays a central role in anabolic signalling pathway emanating from IGF1 through the AKT/S6K pathway, which in turn promotes protein synthesis while suppresses protein degradation through inhibition of FOXO transcription factors regulating MuRF1 and Atrogin‐1.^52^ Several PDK1 substrates, including S6K and protein kinase C, require binding at the PDK1 interacting fragment‐pocket.^53^, ^54^ Small molecule allosteric activators of PDK1 can selectively inhibit activation of substrates that require this docking site interaction.^55^, ^56^ Interestingly, our *in silico* structural modelling predicted that indoprofen might bind to PDK1 in the PDK1 interacting fragment‐pocket (*Figure* 7A). Thus, it is tempting to speculate that indoprofen regulates the substrate specificity of PDK1, thereby leading to anabolic pathway activation. Further studies are required to elucidate the exact molecular mechanisms.

Exercise, and especially resistance exercise, induces muscle adaptation responses such as enhancement of muscle mass and strength, oxidative capacity, mitochondrial protein synthesis,^57^, ^58^ capillary density,^59^ and motor unit recruitment. These events are regulated through sequential time‐dependent changes in molecular responses to resistance exercise.^23^ Upon initiation of resistance exercise, there is an immediate increase in ‘anabolic’ kinase activity, including activation of the AKT/S6K pathway, and following energy consumption redox potentials activate ‘metabolic’ kinases, such as AMPK, that ultimately enhance muscle function through the regulation of mitochondrial protein synthesis and oxidative capacity. Our mechanistic experiments demonstrate that indoprofen evokes a sequential signalling activation resembling the adaptation responses to resistance exercise: an initial activation of the PDK‐AKT‐S6K axis followed by AMPK activation (*Figure* 7C and 7D). Consistent with this model, indoprofen increases muscle mass with enlarged type II fibres and also muscle strength. In alignment with this phenotype, antibody microarrays indicated that the top networks from indoprofen‐treated aged muscles mostly correlated with muscular development, maintenance, and function (*Table* S3). Moreover, in the gene expression profiles of indoprofen‐treated aged muscles, the statistically significant gene ontology (biological process) terms included blood vessel development, extracellular matrix organization, and immune system development (*Figure* 5), which represent muscle adaptation responses to resistance exercise for increasing muscular hypertrophy, strength, power, and local muscular endurance. Thus, indoprofen has a great potential to mimic resistance exercise.

Among the PGC‐1α isoforms, PGC‐1α4 is induced by resistance exercise and evokes muscle hypertrophy through induction of IGF1 and suppression of myostatin.^42^ In the current study, indoprofen treatment elevated PGC‐1α4 expression in muscle, likely contributing to the hypertrophic response (*Figures* 3C–3E and 4K). Recently, a physiological regulation condition that increases PGC‐1α4 expression was reported in suppression of IL6‐ERK‐MAPK inflammation pathway by resistance exercise.^60^ Consistent with this finding, inflammatory regulation has a pleiotropic nature in the regulation of protein balance by promoting both hypertrophic and atrophic programmes in a skeletal muscle.^61^, ^62^, ^63^ Indoprofen, a nonsteroidal anti‐inflammatory drug, has analgesic and anti‐inflammatory properties.^64^ Therefore, this property of indoprofen might be sufficient to induce PGC‐1α4 expression, which in turn contributes to increase in muscle mass independent from PDK1 activation. In atrophic condition, the activation of inflammatory pathways in immobilized muscles^65^ has been suggested to exacerbate muscle damage and loss. Therefore, compounds that counteract inflammation might be beneficial in the treatment of aging‐induced or disuse‐induced atrophy. In support of this hypothesis, our data show that aging‐induced or disuse‐induced muscle wasting was prevented by indoprofen treatment. Several studies have proposed diverse beneficial effects of indoprofen. Indoprofen and its derivatives are involved in the promotion of bone growth or regulation of inflammatory cytokines after exposure to inflammatory stimuli.^64^ Moreover, a previous study has shown that indoprofen enhanced the survival of motor neurons,^35^ which might also contribute to improved muscle strength resembling the exercise effect.

One potential problem of treating human disease with indoprofen is that it was withdrawn from the worldwide market in the 1980s after reports of severe gastrointestinal bleeding. Unlike a dose of 800 mg indoprofen per day, which is known to cause severe conditions, the low concentrations (100 mg, 2 mg/kg) used in our animal experiments did not cause gastrointestinal bleeding or changes in body weight (*Figures* S2A–C, S3A, and S3B). In addition, the hypertrophic response appears to be specific to skeletal muscles, as we did not observe any changes in heart mass (*Figures* 3B and S3C). Indoprofen has relatively lower toxicity in animals (mouse LD_50_: 392 mg/kg) than aspirin (mouse LD_50_: 250 mg/kg), and the latter is commonly used as a safe anti‐inflammatory agent for both acute and long‐term inflammation in humans.^66^ Therefore, it is necessary to set a recommended dosage for use in humans. In addition, the development of indoprofen derivatives with lower gastrointestinal side effects might be effective therapeutics for the treatment of muscle weakness and atrophy. In conclusion, we have demonstrated that indoprofen increases muscle mass in a PDK1‐dependent manner, which in turn improves muscle function through PGC‐1α activation, thus resembling the beneficial effects of resistance exercise.

## Author contributions

S. C. C., H. K., H. J. J., S. C. P., Y. I. L., and J. S. K. developed the study concept and design. H. K., S. C. C., H. J. J., M. H. J., D. R., M. K., Y.S. L., M. S. K., J. H. P., and H. Y. L. performed the experiments and collected the data. H. K., S. C. C., and J. S. K. interpreted the data, prepared figures, and wrote the manuscript.

## Ethical guidelines

The authors certify that they comply with the ethical guidelines for authorship and publishing of the Journal of Cachexia, Sarcopenia and Muscle.^67^

## Conflict of interests

The authors declare no competing interests.

## Supporting information


**Table S1.** The list of primers used qRT‐PCR
**Table S2.** Top‐ranking for indoprofen direct target (PharmMapper)
**Table S3.** Antibody array results from indoprofen treated aging mice muscle

**Figure S1 (A) Experiments performed in triplicate for the top nine compounds PGC‐1α activation.** *n* = 3. (B) qRT‐PCR analysis for expression of PGC‐1α in C2C12 cells treated with the vehicle DMSO or 1, 10, 100 μM indoprofen for 2 days in differentiation medium, *n* = 3. (C) Western blot analysis for PGC‐1α and myoglobin expression in C2C12 cells treated with the vehicle DMSO, 100 μM indoprofen or similar structural drugs (Indomethacin and Ibuprofen) for 2 days in differentiation medium. (D) Quantification of the relative levels of PGC‐1α proteins from panel C, *n* = 3. (E) Quantification of the relative levels of Myoglobin proteins from panel C, *n* = 3. (F) Immunostaining for Myh expression in C2C12 cells treated with vehicle DMSO, indoprofen or similar structural drugs. Scale bar, 25 μm. (G) Quantification of Myh‐positive myotube diameter in panel F. *n* = 4. (H) Western blot analysis for puromycin incorporation in C2C12 cells treated with the vehicle DMSO or 1, 10, 100 μM indoprofen for 2 days in differentiation medium. (I) Quantification of the puromycin incorporation from panel H, *n* = 3. (J) BCA (Bicinchoninic Acid) protein assay for total protein in C2C12 cells treated with the vehicle DMSO, 100 μM indoprofen for 2 days in differentiation medium, *n* = 3. Data are expressed as mean ± SD. To determine statistical significance, an unpaired two‐tailed student t‐test was used (J) and an one‐way ANOVA test with Tukey post‐hoc test was utilized (B, D, E, G, I and J). *p < 0.05 and **p < 0.01 (Indoprofen vs. Control).

**Figure S2 (A) Body weights from control or 2 mg/kg indoprofen ingested 3‐month‐old mice for 4 weeks, *n* = 4.** (B) Food intake from control or 2 mg/kg indoprofen ingested 3‐month‐old mice, *n* = 4. (C) Water intake from control or 2 mg/kg indoprofen ingested 3‐month‐old mice, *n* = 4. (D) Immunostaining of laminin in the TA muscles of control or 2 mg/kg indoprofen‐ingested mice for 4 weeks. Scale bar, 50 μm. (E) Quantification the cross‐sectional area of myofibers in laminin‐positive TA muscles in panel D, *n* = 3. (F, G) Quantification of the overall mean fiber diameters of MyhIIa‐(Control: 263, Indoprofen: 262) and MyhIIb‐(Control: 160, Indoprofen: 169) positive myofibers in panel Figure 2B, *n* = 3. (H) Western blot analysis for expression of PGC‐1α, p‐AMPK and AMPK in muscles from 4‐month‐old mice ingested with control or 2 mg/kg indoprofen for 4 weeks. (I) Quantification of the relative levels of proteins from panel H, *n* = 3. For the calculation of relative phosphorylation levels, the densitometries of the immunoblots of the phospho‐AMPK were normalized to the total AMPK protein levels. Data are expressed as mean ± SD. To determine statistical significance, the student t‐test was used. *p < 0.05, **p < 0.01 and ***p < 0.001 (Indoprofen vs. Control).

**Figure S3 (A) Histology of colon sections stained with hematoxylin and eosin (H&E) from old‐control (25‐month‐old) and old‐indoprofen (2 mg/kg) ingested mice for 12 weeks.** Scale bar, 50 μm. (B) Body weights from control or 2 mg/kg indoprofen ingested 25‐month‐old mice for 12 weeks, *n* = 6. (C) The heart muscle mass of control and 2 mg/kg indoprofen treated 25‐month‐old mice for 12 weeks, *n* = 4. (D) Western blot analysis for expression of total‐OXPHOS in quadriceps muscles from 25‐month‐old mice ingested with control or 2 mg/kg indoprofen for 12 weeks. (E) Quantification of the relative levels of total‐OXPHOS proteins from panel D, *n* = 4. (F) Immunostaining of laminin in the TA muscles of control or 2 mg/kg indoprofen‐ingested mice for 12 weeks. Scale bar, 50 μm. (G) Quantification the cross‐sectional area of myofibers in laminin‐positive TA muscles in panel F, *n* = 3. (H, I) Quantification of the overall mean fiber diameters of MyhIIa‐(Control: 136, Indoprofen: 119) and MyhIIb‐(Control: 100, Indoprofen: 102) positive myofibers in panel Figure 4E, *n* = 3. (J) qRT‐PCR analysis for expression of VEGFα and VEGFβ in quadriceps muscles from control‐ or 2 mg/kg indoprofen‐ingested old mice for 12 weeks, *n* = 4. Data are expressed as mean ± SD. To determine statistical significance, the student t‐test was used. *p < 0.05 and **p < 0.01 (Indoprofen vs. Control).

**Figure S4 (A) Heatmaps showing gene expression patterns of four gene sets (GO_SKELTAL_ SYSTEM_MORPHOGENESIS, GO_MITOCHONDRIAL_GENE_EXPRESSION, and GO_REPONSE_TO_INSULIN).** (B) Western blot analysis of p‐AKT, AKT and Atrogin1 expression in C2C12 cells normal differentiation medium for 1 day and treated with vehicle, indoprofen (100 μM) or dexamethasone (10 μM) alone or in the combination for 2 days. (C) Quantification of the relative protein levels of p‐AKT, AKT and Atrogin1 from panel B, *n* = 3. For the calculation of relative phosphorylation levels, the densitometries of the immunoblots of the phospho‐AKT were normalized to the total AKT protein levels. (D) Hematoxylin and eosin (H&E) staining of TA muscles from control or unloading‐control or unloading‐Indoprofen (2 mg/kg) ingested 3‐month‐old mice for 2 weeks. Scale bar, 25 μm. (E, F) Quantification of the overall mean fiber diameters of MyhIIa‐(Control: 154, Unloading: 189, Unloading+Indoprofen: 197) and MyhIIb‐(Control: 116, Unloading: 104, Unloading+ Indoprofen: 125) positive myofibers in panel Figure 6G, *n* = 3. (G) Immunostaining of laminin in the TA muscles from control or unloading‐control or unloading‐Indoprofen (2 mg/kg) ingested 3‐month‐old mice for 2 weeks. Scale bar, 50 μm. (H) Quantification the cross‐sectional area of myofibers in laminin‐positive TA muscles in panel B, *n* = 3. (I) Twitch isometric force measurement for SOL (left) and EDL (right) muscle function. Data are expressed as mean ± SD. To determine statistical significance, two‐way ANOVA test with Tukey post‐hoc test was utilized. *p < 0.05, **p < 0.01 and ***p < 0.001 (Unloading vs. Control); ^†^ *p* < 0.05, ^††^ *p* < 0.01 and ^†††^ *p* < 0.001 (Unloading+Indoprofen vs. Unloading).

**Figure S5 (A‐C) The relative PGC‐1α‐luciferase activity (2 kb, △Mef2 and △Cre promoter) in C2C12 cells treated with the vehicle DMSO and 100 μM indoprofen for 2 days, *n* = 3.** (D) The relative PGC‐1α‐luciferase activity (2 kb, △Mef2, △Cre promoter) of C2C12 cells treated with the vehicle DMSO or indoprofen (100 μM) with or without GSK2334470 (3 μM) for 4 hours in stabilized differentiation medium condition, *n* = 3. (E) The relative PGC‐1α‐luciferase activity (2 kb, △Mef2 and △Cre promoter) of C2C12 cells treated with vehicle, indoprofen (100 μM) or Compound C (2 μM) alone or in the combination for 24 hours in differentiation medium, *n* = 3. Data are expressed as mean ± SD. To determine statistical significance, an unpaired two‐tailed student t‐test was used (A‐C) and two‐way ANOVA test with Tukey post‐hoc analysis (D and E) was utilized. *p < 0.05, **p < 0.01 and ***p < 0.001 (Indoprofen, PDK1 inhibitor or Compound C vs. Control); ^†^ *p* < 0.05 and ^††^ *p* < 0.01 (PDK1 inhibitor+Indoprofen or Compound C + Indoprofen vs. PDK1 inhibitor or Compound C).

**Figure S6 (A) Coomassie staining of the purified MBP‐tagged PDK1 protein (line 1 and 4 = size marker, line 2 = commercial PDK1, line 3 = in‐house purified MBP‐tagged PDK1 protein).** (B) The test of reaction time optimized in indicated times (10‐60 minutes) using *in vitro* protein kinase assay, *n* = 3. (C) Biacore SPR assay of the interaction between PDK1 and various concentration of indoprofen. (D) qRT‐PCR analysis for expression of arachidonic acid‐related genes, PLA2G5, PTGES2 and PTGS1 in C2C12 cells treated with the vehicle DMSO or 100 μM indoprofen for 2 days in differentiation medium, *n* = 3. (E) Western blot analysis for expression of p‐Tyr, p‐IRS, IRS, p‐PI3K(p85), p‐PI3K(p55) and PI3K of C2C12 cells treated with vehicle or indoprofen (100 μM) for 10 minutes, 2 hours, 4 hours and 6 hours in stabilized differentiation medium condition. (F) Quantification of the relative levels of proteins from panel E, *n* = 3. (G) Western blot analysis for PGC‐1α, total‐OXPHOS, p‐PDK1 and PDK1, of C2C12 cells transfected lentiviral PDK1 knockdown treated with vehicle or indoprofen (100 μM) for 24 hours in stabilized differentiation medium condition. (H) Quantification of the relative levels of PGC‐1α and total‐OXPHOS proteins from panel G, *n* = 3. (I) Quantification of the overall mean fiber diameters of MyhIIa‐(Control: 142, shPDK1: 143, shPDK1 + Indoprofen: 125) and MyhIIb‐(Control: 169, shPDK1: 162, shPDK1 + Indoprofen: 159) positive myofibers in panel Figure 8B, *n* = 3. (J) Immunostaining of laminin in the TA muscles of control, shPDK1 or shPDK1 + indoprofen (2 mg/kg, for 2 weeks), for ‐ingested mice. Scale bar, 50 μm. (K) Quantification the cross‐sectional area of myofibers in laminin‐positive TA muscles in panel K, *n* = 3. For the calculation of relative phosphorylation levels, the densitometries of the immunoblots of the phospho‐Abs were normalized to the total protein levels. Data are expressed as mean ± SD. To determine statistical significance, an unpaired two‐tailed student t‐test was used (D and F) and two‐way ANOVA test with Tukey post‐hoc analysis was utilized (H, I and K). *p < 0.05, **p < 0.01 and ***p < 0.001 (Indoprofen or shPDK1 vs. Control); ^††^ *p* < 0.01 and ^†††^ *p* < 0.001 (PDK1 + Indoprofen vs. PDK1).
