# Supplementary material for: Indoprofen prevents muscle wasting in aged mice through activation of PDK1/AKT pathway
Source: J Cachexia Sarcopenia Muscle. 2020 Feb 25;11(4):1070–88. doi: 10.1002/jcsm.12558 (PMC7432593; doi:10.1002/jcsm.12558)
Supplement: Supplementary file 1 — Table S1. The list of primers used qRT‐PCR Table S2. Top‐ranking for indoprofen direct target (PharmMapper) Table S3. Antibody array results from indoprofen treated aging mice muscle [file JCSM-11-1070-s001.pdf]

Supplementary table 1.

The list of primers used qRT-PCR

Target Genes		Mouse Primers (5' to 3')
**Total PGC-1α**	Forward	TGATGTGAATGACTTGGATACAGACA
	Reverse	GCTCATTGTTGTACTGGTTGGATATG
**PGC-1α1**	Forward	GGACATGTGCAGCCAAGACTCT
	Reverse	CACTTCAATCCACCCAGAAAGCT
**PGC-1α2**	Forward	CCACCAGAATGAGTGACATGGA
	Reverse	GTTCAGCAAGATCTGGGCAAA
**PGC-1α3**	Forward	AAGTGAGTAACCGGAGGCATTC
	Reverse	TTCAGGAAGATCTGGGCAAAGA
**PGC-1α4**	Forward	TCACACCAAACCCACAGAAA
	Reverse	CTGGAAGATATGGCACAT
**Atrogin-1**	Forward	CAACATTAACATGTGGGTGTAT
	Reverse	GTCACTCAGCCTCTGCATG
**MuRF1**	Forward	GAGAACCTGGAGAAGCAGCT
	Reverse	CCGCGGTTGGTCCAGTAG
**VEGFα**	Forward	ACAGCACAGCAGATGTGAAT
	Reverse	ACAGTGAACGCTCCAGGATT
**VEGFβ**	Forward	AAGCCAGACAGGGTTGCCAT
	Reverse	TGGATGATGTCAGCTGGGGAG
**L32**	Forward	GGCCTCTGGTGAAGCCCAAGATCG
	Reverse	CCTCTGGGTTTCCGCCAGTTTCGC
**PLA2G5**	Forward	GGGGTGTCAAGGAAGAAGAGGA
	Reverse	CTTGGGTTCTTTGTAGCCTGGTC
**PTGES2**	Forward	CATCAGCAAGCGCCTCAAAA
	Reverse	CATACACTGCCAGGTCAGCA
**PTGS1**	Forward	GGCATTGCACATCCATCCAC
	Reverse	CAGGGATTGACTGGTGAGGG

Supplementary table 2.

Top-ranking for indoprofen direct target (PharmMapper)

Result of 160929153136

Top 300 targets ranked by fit score in descending order

Ligand: INDOPROFEN								
	Rank	PDB ID	Target Name	Number of Feature	Fit Score	Normalized Fit Score	z'-score
+	1	2PE1	3-phosphoinositide-dependent protein kinase 1	7	4.562	0.6517	1.93902
+	2	1QXO	Chorismate synthase	10	4.437	0.4437	0.766297
+	3	1UBW	Farnesyl pyrophosphate synthetase	9	4.225	0.4694	1.3833
+	4	1R1H	Neprilysin	10	4.204	0.4204	0.807085

Supplementary table 3.

Antibody array results from indoprofen treated aging mice muscle

Top Networks

ID	Associated Network Functions	Score
1	Cellular Assembly and Organization, Cellular Function and Maintenance, Skeletal and Muscular System Development and Function	47
2	Cellular Development, Hematological System Development and Function, Hematopoiesis	32
3	Cell-To-Cell Signaling and Interaction, Cellular Assembly and Organization, Cellular Function and Maintenance	28
4	Cancer, Embryonic Development, Cellular Development	24
5	Molecular Transport, Cell Morphology, Cellular Function and Maintenance	20

Top Diseases and Bio Functions

Molecular and Cellular Functions

Name	p-value	#Molecules
Cellular Assembly and Organization	1.74E-02 - 1.15E-05	12
Cellular Function and Maintenance	1.91E-02 - 1.15E-05	25
Cell Death and Survival	1.74E-02 - 6.52E-04	22
Cellular Development	1.95E-02 - 6.52E-04	22
Cell-To-Cell Signaling and Interaction	1.99E-02 - 6.91E-04	22
