# Supplementary figures and images for: Indoprofen prevents muscle wasting in aged mice through activation of PDK1/AKT pathway
Source: J Cachexia Sarcopenia Muscle. 2020 Feb 25;11(4):1070–88. doi: 10.1002/jcsm.12558 (PMC7432593; doi:10.1002/jcsm.12558)

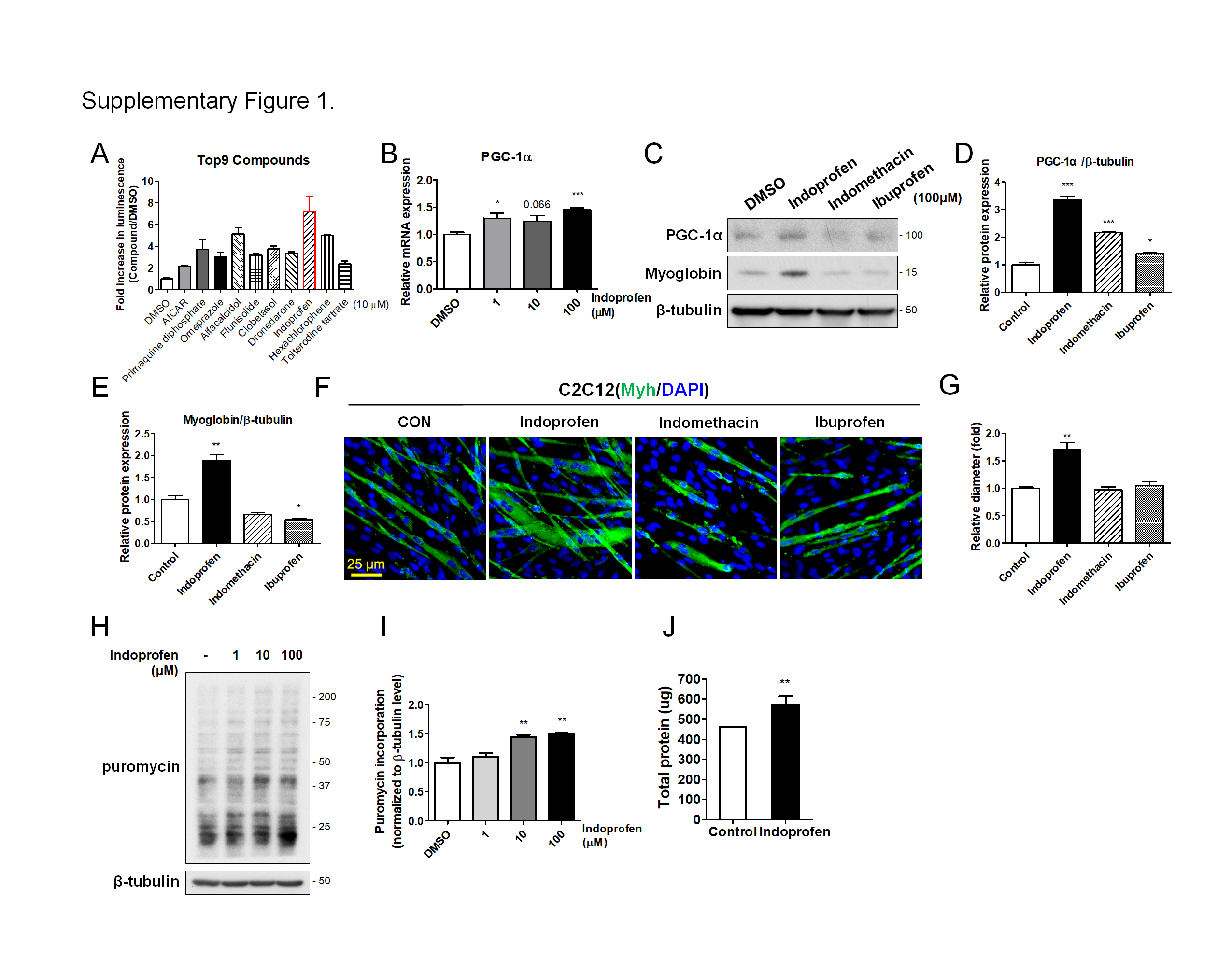

Supplement: Supplementary file 2 — Figure S1 (A) Experiments performed in triplicate for the top nine compounds PGC‐1α activation. n = 3. (B) qRT‐PCR analysis for expression of PGC‐1α in C2C12 cells treated with the vehicle DMSO or 1, 10, 100 μM indoprofen for 2 days in differentiation medium, n = 3. (C) Western blot analysis for PGC‐1α and myoglobin expression in C2C12 cells treated with the vehicle DMSO, 100 μM indoprofen or similar structural drugs (Indomethacin and Ibuprofen) for 2 days in differentiation medium. (D) Quantification of the relative levels of PGC‐1α proteins from panel C, n = 3. (E) Quantification of the relative levels of Myoglobin proteins from panel C, n = 3. (F) Immunostaining for Myh expression in C2C12 cells treated with vehicle DMSO, indoprofen or similar structural drugs. Scale bar, 25 μm. (G) Quantification of Myh‐positive myotube diameter in panel F. n = 4. (H) Western blot analysis for puromycin incorporation in C2C12 cells treated with the vehicle DMSO or 1, 10, 100 μM indoprofen for 2 days in differentiation medium. (I) Quantification of the puromycin incorporation from panel H, n = 3. (J) BCA (Bicinchoninic Acid) protein assay for total protein in C2C12 cells treated with the vehicle DMSO, 100 μM indoprofen for 2 days in differentiation medium, n = 3. Data are expressed as mean ± SD. To determine statistical significance, an unpaired two‐tailed student t‐test was used (J) and an one‐way ANOVA test with Tukey post‐hoc test was utilized (B, D, E, G, I and J). *p < 0.05 and **p < 0.01 (Indoprofen vs. Control). [file JCSM-11-1070-s002.tif]

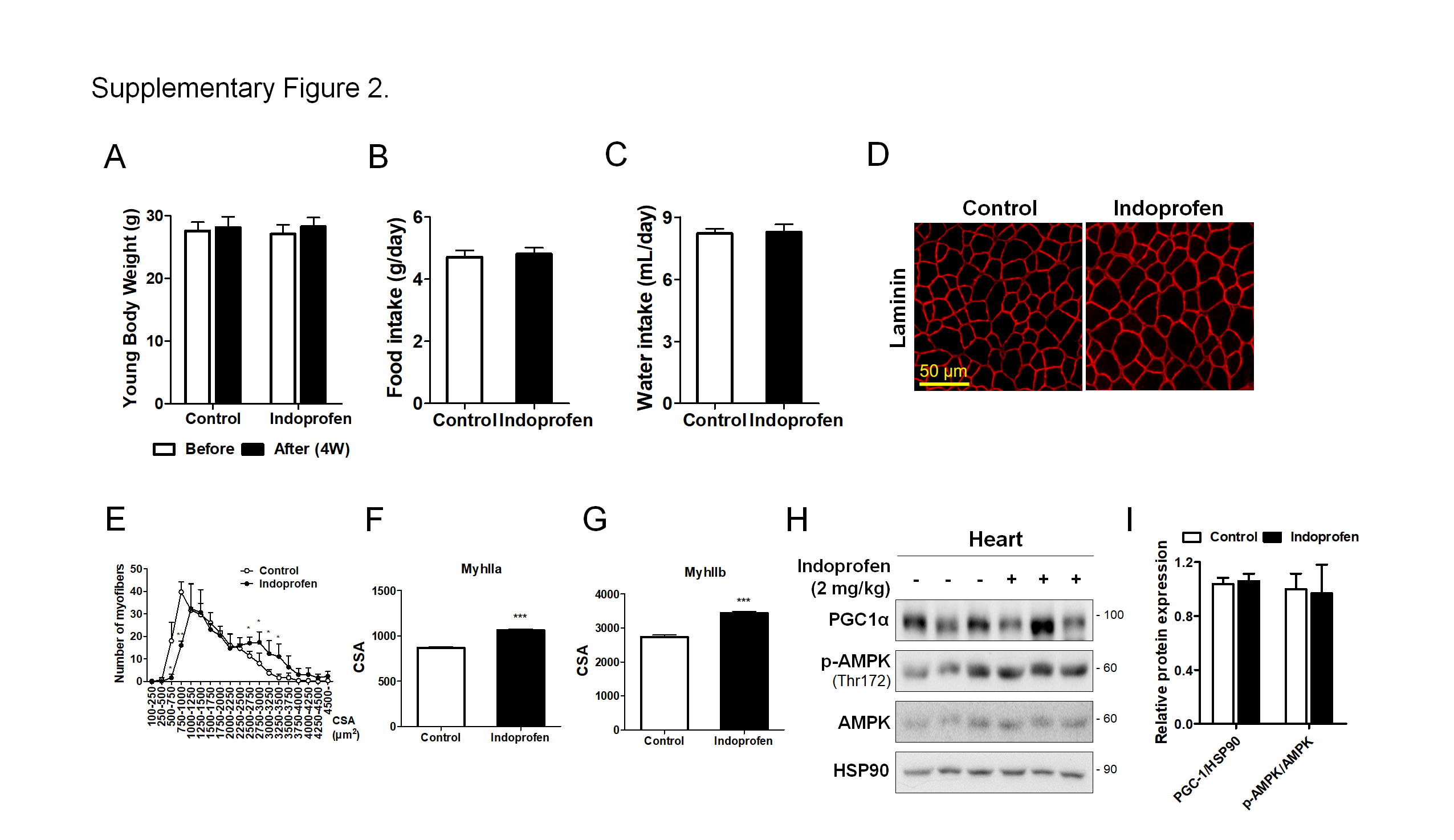

Supplement: Supplementary file 3 — Figure S2 (A) Body weights from control or 2 mg/kg indoprofen ingested 3‐month‐old mice for 4 weeks, n = 4. (B) Food intake from control or 2 mg/kg indoprofen ingested 3‐month‐old mice, n = 4. (C) Water intake from control or 2 mg/kg indoprofen ingested 3‐month‐old mice, n = 4. (D) Immunostaining of laminin in the TA muscles of control or 2 mg/kg indoprofen‐ingested mice for 4 weeks. Scale bar, 50 μm. (E) Quantification the cross‐sectional area of myofibers in laminin‐positive TA muscles in panel D, n = 3. (F, G) Quantification of the overall mean fiber diameters of MyhIIa‐(Control: 263, Indoprofen: 262) and MyhIIb‐(Control: 160, Indoprofen: 169) positive myofibers in panel Figure 2B, n = 3. (H) Western blot analysis for expression of PGC‐1α, p‐AMPK and AMPK in muscles from 4‐month‐old mice ingested with control or 2 mg/kg indoprofen for 4 weeks. (I) Quantification of the relative levels of proteins from panel H, n = 3. For the calculation of relative phosphorylation levels, the densitometries of the immunoblots of the phospho‐AMPK were normalized to the total AMPK protein levels. Data are expressed as mean ± SD. To determine statistical significance, the student t‐test was used. *p < 0.05, **p < 0.01 and ***p < 0.001 (Indoprofen vs. Control). [file JCSM-11-1070-s003.tif]

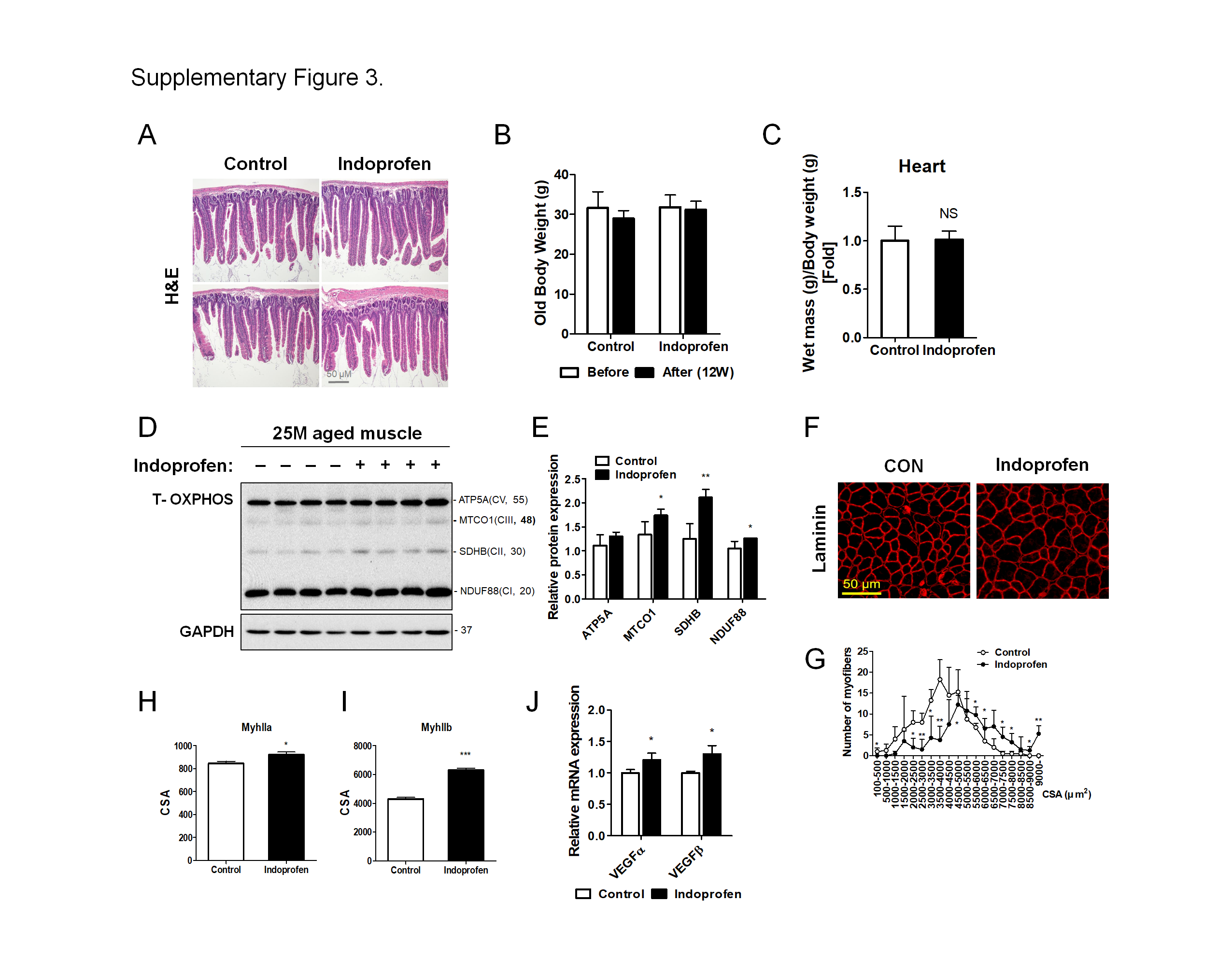

Supplement: Supplementary file 4 — Figure S3 (A) Histology of colon sections stained with hematoxylin and eosin (H&E) from old‐control (25‐month‐old) and old‐indoprofen (2 mg/kg) ingested mice for 12 weeks. Scale bar, 50 μm. (B) Body weights from control or 2 mg/kg indoprofen ingested 25‐month‐old mice for 12 weeks, n = 6. (C) The heart muscle mass of control and 2 mg/kg indoprofen treated 25‐month‐old mice for 12 weeks, n = 4. (D) Western blot analysis for expression of total‐OXPHOS in quadriceps muscles from 25‐month‐old mice ingested with control or 2 mg/kg indoprofen for 12 weeks. (E) Quantification of the relative levels of total‐OXPHOS proteins from panel D, n = 4. (F) Immunostaining of laminin in the TA muscles of control or 2 mg/kg indoprofen‐ingested mice for 12 weeks. Scale bar, 50 μm. (G) Quantification the cross‐sectional area of myofibers in laminin‐positive TA muscles in panel F, n = 3. (H, I) Quantification of the overall mean fiber diameters of MyhIIa‐(Control: 136, Indoprofen: 119) and MyhIIb‐(Control: 100, Indoprofen: 102) positive myofibers in panel Figure 4E, n = 3. (J) qRT‐PCR analysis for expression of VEGFα and VEGFβ in quadriceps muscles from control‐ or 2 mg/kg indoprofen‐ingested old mice for 12 weeks, n = 4. Data are expressed as mean ± SD. To determine statistical significance, the student t‐test was used. *p < 0.05 and **p < 0.01 (Indoprofen vs. Control). [file JCSM-11-1070-s004.tif]

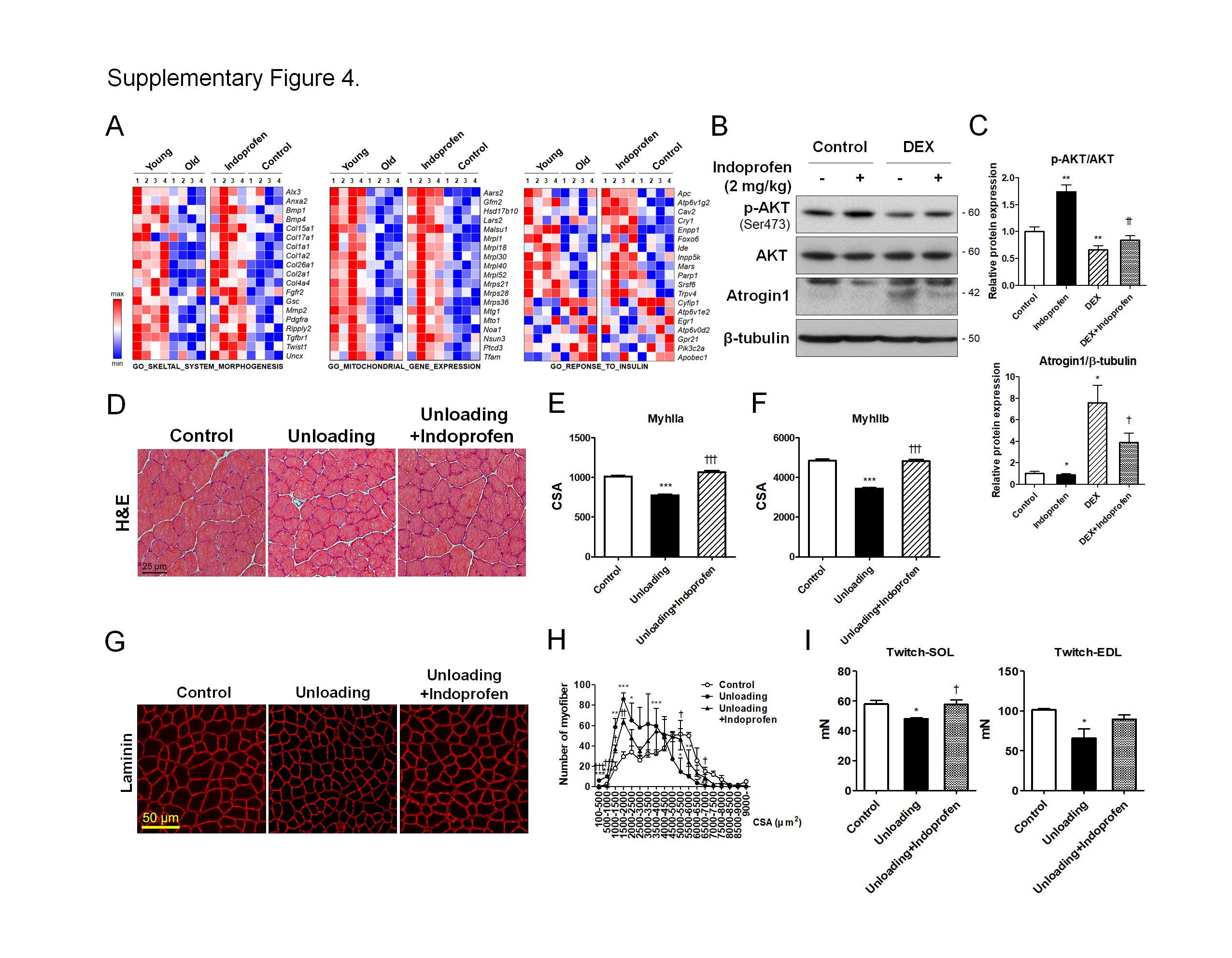

Supplement: Supplementary file 5 — Figure S4 (A) Heatmaps showing gene expression patterns of four gene sets (GO_SKELTAL_ SYSTEM_MORPHOGENESIS, GO_MITOCHONDRIAL_GENE_EXPRESSION, and GO_REPONSE_TO_INSULIN). (B) Western blot analysis of p‐AKT, AKT and Atrogin1 expression in C2C12 cells normal differentiation medium for 1 day and treated with vehicle, indoprofen (100 μM) or dexamethasone (10 μM) alone or in the combination for 2 days. (C) Quantification of the relative protein levels of p‐AKT, AKT and Atrogin1 from panel B, n = 3. For the calculation of relative phosphorylation levels, the densitometries of the immunoblots of the phospho‐AKT were normalized to the total AKT protein levels. (D) Hematoxylin and eosin (H&E) staining of TA muscles from control or unloading‐control or unloading‐Indoprofen (2 mg/kg) ingested 3‐month‐old mice for 2 weeks. Scale bar, 25 μm. (E, F) Quantification of the overall mean fiber diameters of MyhIIa‐(Control: 154, Unloading: 189, Unloading+Indoprofen: 197) and MyhIIb‐(Control: 116, Unloading: 104, Unloading+ Indoprofen: 125) positive myofibers in panel Figure 6G, n = 3. (G) Immunostaining of laminin in the TA muscles from control or unloading‐control or unloading‐Indoprofen (2 mg/kg) ingested 3‐month‐old mice for 2 weeks. Scale bar, 50 μm. (H) Quantification the cross‐sectional area of myofibers in laminin‐positive TA muscles in panel B, n = 3. (I) Twitch isometric force measurement for SOL (left) and EDL (right) muscle function. Data are expressed as mean ± SD. To determine statistical significance, two‐way ANOVA test with Tukey post‐hoc test was utilized. *p < 0.05, **p < 0.01 and ***p < 0.001 (Unloading vs. Control); † p < 0.05, †† p < 0.01 and ††† p < 0.001 (Unloading+Indoprofen vs. Unloading). [file JCSM-11-1070-s005.tif]

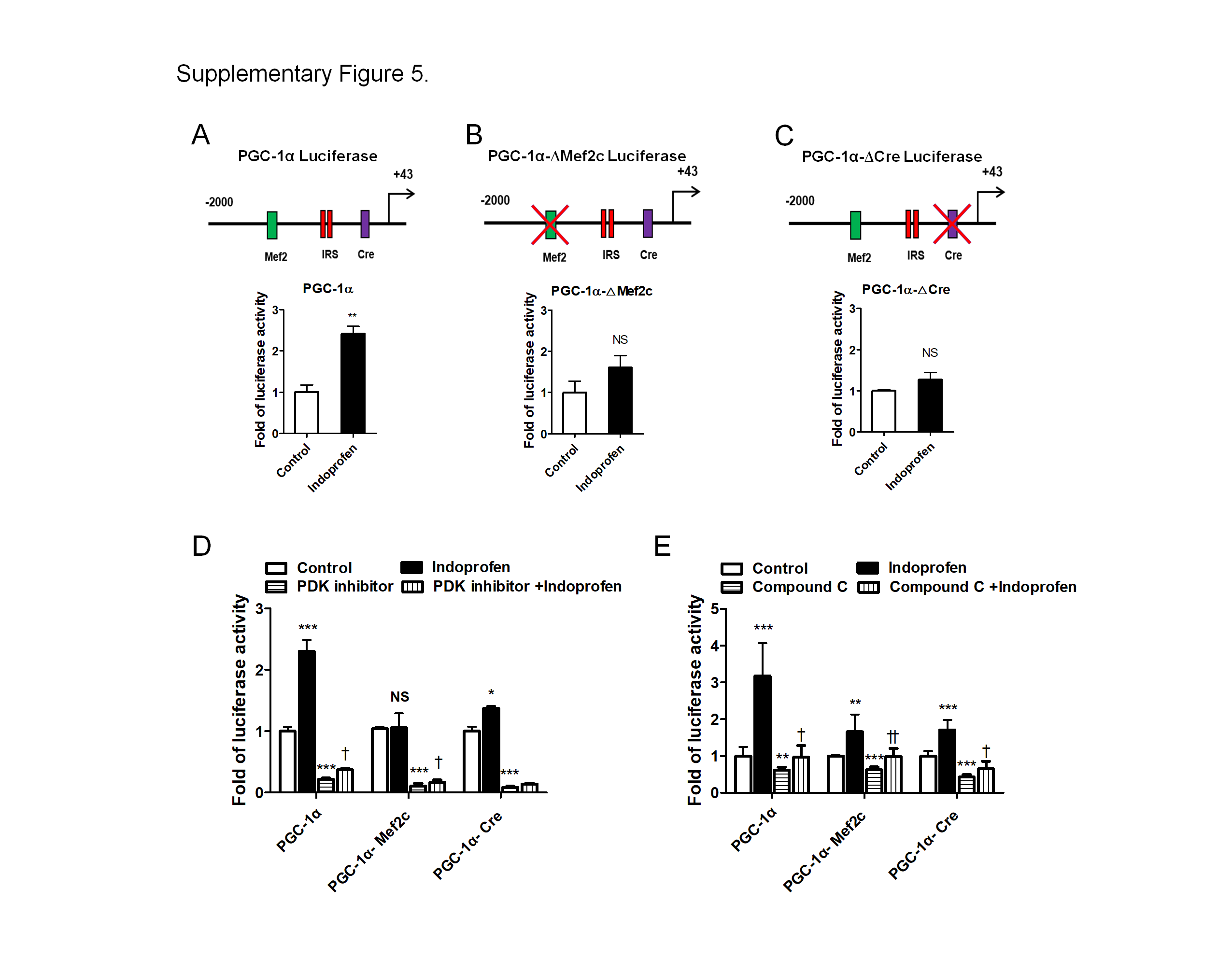

Supplement: Supplementary file 6 — Figure S5 (A‐C) The relative PGC‐1α‐luciferase activity (2 kb, △Mef2 and △Cre promoter) in C2C12 cells treated with the vehicle DMSO and 100 μM indoprofen for 2 days, n = 3. (D) The relative PGC‐1α‐luciferase activity (2 kb, △Mef2, △Cre promoter) of C2C12 cells treated with the vehicle DMSO or indoprofen (100 μM) with or without GSK2334470 (3 μM) for 4 hours in stabilized differentiation medium condition, n = 3. (E) The relative PGC‐1α‐luciferase activity (2 kb, △Mef2 and △Cre promoter) of C2C12 cells treated with vehicle, indoprofen (100 μM) or Compound C (2 μM) alone or in the combination for 24 hours in differentiation medium, n = 3. Data are expressed as mean ± SD. To determine statistical significance, an unpaired two‐tailed student t‐test was used (A‐C) and two‐way ANOVA test with Tukey post‐hoc analysis (D and E) was utilized. *p < 0.05, **p < 0.01 and ***p < 0.001 (Indoprofen, PDK1 inhibitor or Compound C vs. Control); † p < 0.05 and †† p < 0.01 (PDK1 inhibitor+Indoprofen or Compound C + Indoprofen vs. PDK1 inhibitor or Compound C). [file JCSM-11-1070-s006.tif]

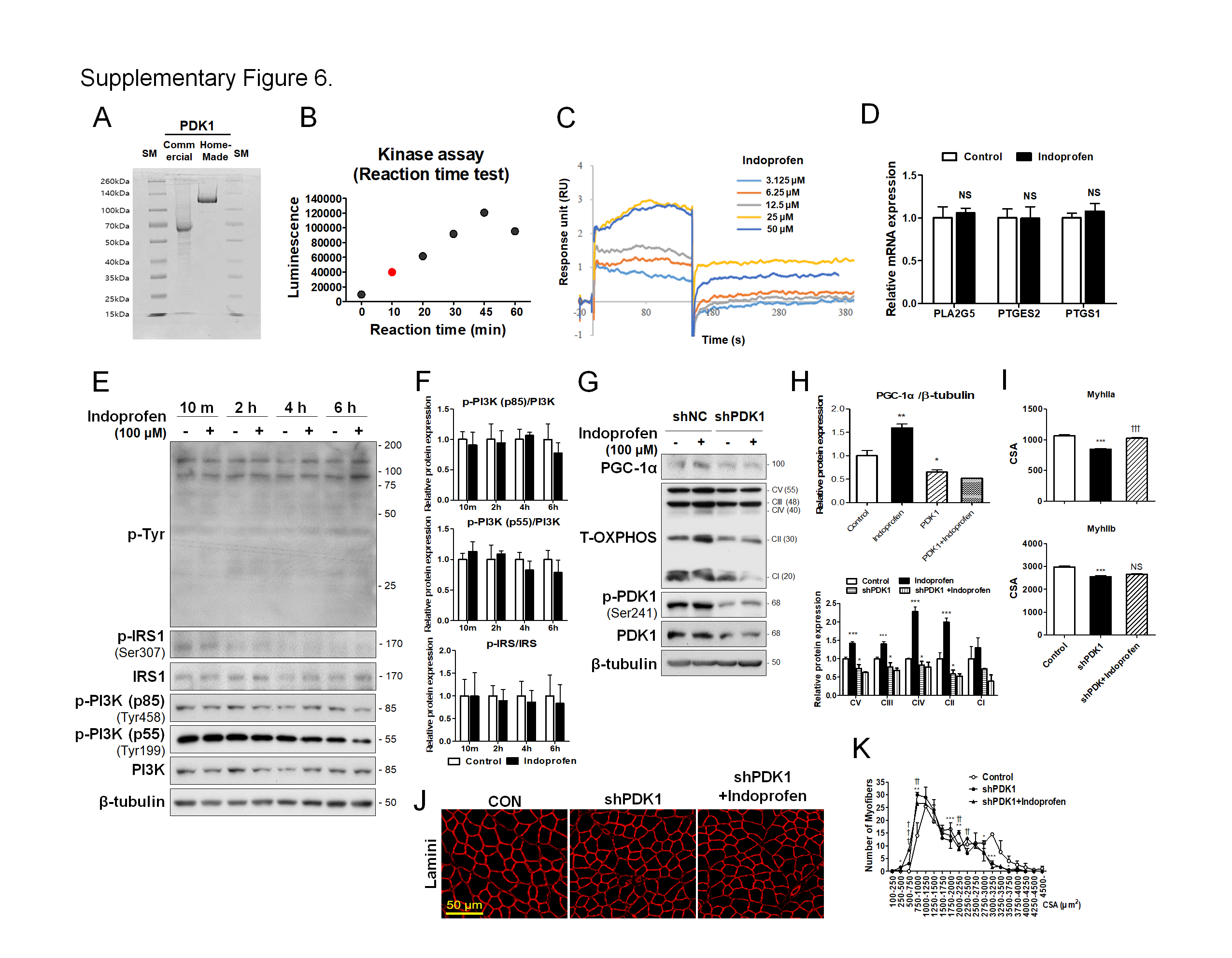

Supplement: Supplementary file 7 — Figure S6 (A) Coomassie staining of the purified MBP‐tagged PDK1 protein (line 1 and 4 = size marker, line 2 = commercial PDK1, line 3 = in‐house purified MBP‐tagged PDK1 protein). (B) The test of reaction time optimized in indicated times (10‐60 minutes) using in vitro protein kinase assay, n = 3. (C) Biacore SPR assay of the interaction between PDK1 and various concentration of indoprofen. (D) qRT‐PCR analysis for expression of arachidonic acid‐related genes, PLA2G5, PTGES2 and PTGS1 in C2C12 cells treated with the vehicle DMSO or 100 μM indoprofen for 2 days in differentiation medium, n = 3. (E) Western blot analysis for expression of p‐Tyr, p‐IRS, IRS, p‐PI3K(p85), p‐PI3K(p55) and PI3K of C2C12 cells treated with vehicle or indoprofen (100 μM) for 10 minutes, 2 hours, 4 hours and 6 hours in stabilized differentiation medium condition. (F) Quantification of the relative levels of proteins from panel E, n = 3. (G) Western blot analysis for PGC‐1α, total‐OXPHOS, p‐PDK1 and PDK1, of C2C12 cells transfected lentiviral PDK1 knockdown treated with vehicle or indoprofen (100 μM) for 24 hours in stabilized differentiation medium condition. (H) Quantification of the relative levels of PGC‐1α and total‐OXPHOS proteins from panel G, n = 3. (I) Quantification of the overall mean fiber diameters of MyhIIa‐(Control: 142, shPDK1: 143, shPDK1 + Indoprofen: 125) and MyhIIb‐(Control: 169, shPDK1: 162, shPDK1 + Indoprofen: 159) positive myofibers in panel Figure 8B, n = 3. (J) Immunostaining of laminin in the TA muscles of control, shPDK1 or shPDK1 + indoprofen (2 mg/kg, for 2 weeks), for ‐ingested mice. Scale bar, 50 μm. (K) Quantification the cross‐sectional area of myofibers in laminin‐positive TA muscles in panel K, n = 3. For the calculation of relative phosphorylation levels, the densitometries of the immunoblots of the phospho‐Abs were normalized to the total protein levels. Data are expressed as mean ± SD. To determine statistical significance, an unpaired two‐tailed studen [file JCSM-11-1070-s007.tif]
